# Flexibility of the Rotavirus NSP2 C-terminal Region Supports Factory Formation via Liquid-Liquid Phase Separation

**DOI:** 10.1128/jvi.00039-23

**Published:** 2023-02-07

**Authors:** Sarah L. Nichols, Emil M. Nilsson, Heather Brown-Harding, Leslie E.W. LaConte, Julia Acker, Alexander Borodavka, Sarah McDonald Esstman

**Affiliations:** 1 Department of Biology, Wake Forest University, Winston-Salem, NC; 2 Fralin Biomedical Research Institute, Roanoke, VA; 3 Department of Basic Science Education, Virginia Tech Carilion School of Medicine, Roanoke, VA; 4 Department of Biochemistry, University of Cambridge, Cambridge, UK

**Keywords:** viral factory, LLPS, rotavirus, viroplasm, NSP2, NSP5

## Abstract

Many viruses sequester the materials needed for their replication into discrete subcellular factories. For rotaviruses (RVs), these factories are called viroplasms, and they are formed in the host cell cytosol via the process of liquid-liquid phase separation (LLPS). The non-structural protein 2 (NSP2) and its binding partner non-structural protein 5 (NSP5) are critical for viroplasm biogenesis. Yet, it is not fully understood how NSP2 and NSP5 cooperate to form factories. The C-terminal region of NSP2 (CTR; residues 291-317) is flexible, allowing it to participate in domain-swapping interactions that promote inter-octamer interactions and presumably viroplasm formation. Molecular dynamics simulations showed that a lysine-to-glutamic acid change at position 294 (K294E) reduces NSP2 CTR flexibility *in silico*. To test the impact of reduced NSP2 CTR flexibility during infection, we engineered a mutant RV bearing this change (rRV-NSP2_K294E_). Single-cycle growth assays revealed a >1.2-log reduction in endpoint titers for rRV-NSP2_K294E_ versus wildtype control (rRV-WT). Using immunofluorescence assays, we found that rRV-NSP2_K294E_ formed smaller, more numerous viroplasms as compared to rRV-WT. Live-cell imaging experiments confirmed these results and revealed that rRV-NSP2_K294E_ factories had delayed fusion kinetics. Moreover, NSP2_K294E_ and several other CTR mutants formed fewer viroplasm-like structures in NSP5 co-expressing cells than did control NSP2_WT_. Finally, NSP2_K294E_ exhibited defects in its capacity to induce LLPS droplet formation *in vitro* when incubated alongside NSP5. These results underscore the importance of NSP2 CTR flexibility in supporting the biogenesis of RV factories.

## Introduction

Rotaviruses (RVs) are 11-segmented, double-stranded (ds) RNA viruses in the *Reoviridae* family (1). The RV virion is a non-enveloped, triple-layered particle comprised of 6 structural proteins (VP1-VP4, VP6, and VP7) (2, 3). An additional 6 non-structural proteins (NSP1-NSP6) are also expressed from the genome and play various roles during intracellular replication (1). RV infection is initiated following entry of the virion into the cell and shedding of the outer capsid layer to form a double-layered particle (DLP) (2). The incoming DLP functions as the RV transcriptase complex in the cell cytosol (4). Specifically, VP1 polymerases within the DLP synthesize 11 single-stranded, positive-sense (+) RNAs using the dsRNA genome segments as templates (5, 6). These +RNAs are extruded from aqueous channels that permeate the DLP capsid layers, and they go on to serve as messenger RNA templates for protein synthesis (4, 6). During early virion assembly, the 11 +RNA transcripts are also selectively assorted and packaged into new particles, wherein they are used as templates by VP1 for genome replication (i.e., dsRNA synthesis) (7, 8). Nascent DLPs formed via this assembly process are transcriptionally active, thereby amplifying +RNA and protein levels in the infected cells and ensuring robust RV replication (9). DLPs go on to acquire an outer capsid layer during the final stages of virion morphogenesis in the endoplasmic reticulum (ER), forming a mature virion that exits the cell (2).

Like many other RNA viruses, RVs sequester and concentrate the materials needed for their replication into discrete factories in the cell cytoplasm (10). For RVs, these factories are protein- and RNA-rich inclusion bodies called viroplasms, and they are the sites of DLP assembly and most viral RNA synthesis (i.e., dsRNA synthesis and secondary +RNA transcription) (11). When viewed by electron microscopy (EM) in thin-sectioned, negatively-stained RV-infected cells, viroplasms are seen as electron-dense, non-membrane bound structures that can range in size from <0.1 to >5.0 μm in diameter (11–13). However, because they are so electron-dense, the viral assembly intermediates within viroplasms cannot be resolved using EM (11, 14, 15). When RV-infected cells are stained with fluorescently conjugated antisera against viroplasm resident proteins (i.e., VP1, VP2, VP3, VP6, NSP2, and NSP5) and viewed by immunofluorescence microscopy, the factories can be seen as punctate, spherical cytoplasmic foci starting around 2-4 h post-infection (p.i.). Viroplasms begin relatively small (~0.1 to 1.0 μm in diameter), but they increase in size as infection proceeds, with some reaching >5.0 μm in diameter by 12-24 h p.i. (13). The increase in viroplasm size was shown to correlate with a decrease in the number of viroplasms per cell, leading to the hypothesis that these factories are dynamic and fuse together over time (13). Live-cell imaging of RV-infected cells expressing fluorescent-fusion versions of either NSP2 or NSP5 support this notion, revealing liquid-like viroplasmic spheres that merge over time (13, 16). Even more, Geiger et al. recently showed that early-stage viroplasms formed ~2-6 h p.i. are sensitive to dissolution by aliphatic diols, indicating that factory biogenesis involves the process of liquid-liquid phase separation (LLPS) (16).

While several viral and host cell proteins have been shown to associate with viroplasms, RV non-structural proteins NSP2 and NSP5 are largely considered to be the main drivers of factory nucleation (11, 17–19). Ectopic expression of these binding partners alone is sufficient to drive the formation of viroplasm-like structures (VLS) in the cell cytosol (20–23). Moreover, purified recombinant NSP2 and NSP5 proteins interact and undergo rapid condensation *in vitro,* forming viroplasm-like LLPS droplets (16). NSP5 is a 22-kDa, serine/threonine-rich, intrinsically disordered protein that can assemble into several higher-ordered oligomers (24). During infection, this protein is also O-glycosylated and differentially phosphorylated, causing it to migrate in sodium dodecyl sulfate (SDS)-polyacrylamide gels as isoforms ranging in size from 26 to 35 kDa (25–27). Phosphorylation of NSP5 is required for proper viroplasm morphology and for viral replication, but not for VLS formation or LLPS (28). NSP5 directly interacts with NSP2, a 35-kDa protein that self-assembles into a donut-shaped octamer (29). As a recombinant octameric protein, NSP2 has several *in vitro* activities, including RNA helix destabilizing, RNA-RNA chaperone, nucleoside triphosphatase (NTPase), RNA triphosphatase (RTPase), and nucleoside diphosphate (NDP) kinase (30–34). A histidine-to-alanine change at NSP2 position 225 (H225A) abolishes NTPase/RTPase activity, but it does not prevent viroplasm formation, suggesting that these functions of the protein are distinct (33).

**Fig. 1:**
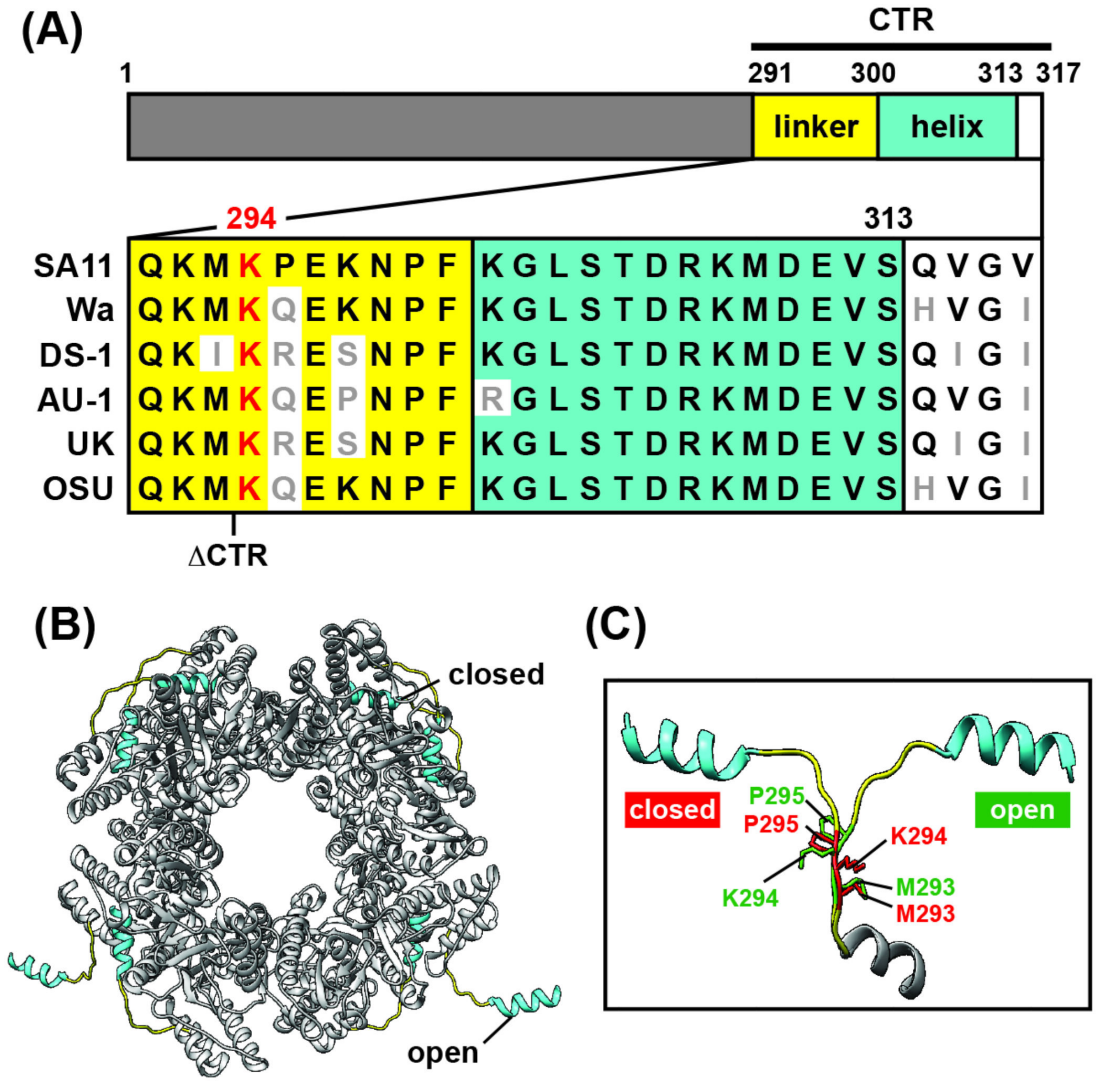
Sequence conservation and structure of NSP2 CTR. (A) Cartoon schematic of NSP2 (317 amino acids in length). The core of the protein is made up of residues 1-290 (grey). The C-terminal region (CTR) is comprised of a linker (yellow; residues 291-300), a C-terminal a-helix (cyan; residues 301-313), and an unstructured region (white; residues 314-317). An amino acid sequence alignment of the NSP2 CTR from a variety of human and animal strains is shown below the schematic. Non-conserved residues are colored grey and lysine 294 is colored red. The truncation location of a previously described CTR mutant (ΔCTR, missing residues 293-317) is indicated. (B) The octameric structure of strain SA11 NSP2 modeled in Chimera software (PDB#4G0A) is colored as in panel A (32). The CTR exhibits open (domain-swapping) and closed (non-domaining swapping) conformations. (C) Zoom view of super-positioned, open and closed conformations of the NSP2 CTR. Linker residues M293, K294, and P295 are shown in stick representation and labeled (open residues are colored green, and closed residues are colored red).

The extreme C-terminal region of NSP2 (CTR; residues ~291-317) is comprised of a flexible linker domain (residues 291-300) that connects a C-terminal helix (residues 301-313) to the main NSP2 core (residues 1-290) (Fig. 1A) (29, 32). The NSP2 CTR plays a critical albeit poorly understood role in viroplasm biogenesis. In the context of an infected cell, serine 313 within the NSP2 CTR can be phosphorylated, and a mutant RV bearing a phosphomimetic S313D change exhibits defects in viroplasm formation (12, 35). However, like what is seen for NSP5, the phosphorylation of NSP2 is not essential for the formation of VLS in cells or LLPS condensates *in vitro*. Still, deletion of CTR residues 293-317 was shown to hamper VLS formation in NSP5 co-expressing monkey kidney MA104 cells, implicating its critical role in factory biogenesis in a manner distinct from phosphorylation (36). Under crystallographic conditions, the flexible NSP2 CTR was shown to participate in domain-swapping interactions that link several NSP2 octamers together, a feature that is hypothesized to facilitate viroplasm formation (32). Flexibility of the NSP2 CTR, a prerequisite for inter-octamer interactions, is dictated in part by a highly conserved lysine residue at position 294 (Fig. 1A). Specifically, the NSP2 CTR can be seen in either an open (i.e., domain-swapping) or a closed (i.e., non-domain swapping) state. The open versus closed states correlate with large movements in a highly conserved lysine residue at position 294 (K294), while neighboring residues barely shift (Fig. 1B and 1C). Thus, K294 is expected to be a key determinant of NSP2 CTR flexibility and viroplasm biogenesis. In the current study, we tested this notion using a combination of computational, genetic, and biochemical approaches. In particular, we show that a lysine to asparatic acid change at position 294 (K294E): (i) reduces NSP2 CTR flexibility *in silico,* (ii) causes RV to form smaller, more numerous viroplasms during infection, (iii) decreases viroplasm fusion kinetics, (iv) reduces the number of VLS in NSP2/NSP5-co-expressing cells, and (v) mitigates the capacity of NSP2 and NSP5 to undergo LLPS *in vitro*. Thus, we provide strong evidence to support an important role for NSP2 CTR flexibility during rotavirus factory formation, thereby adding to our textbook knowledge of this virus.

## Results

### A K294E amino acid change decreases NSP2 CTR flexibility *in silico*. The NSP2

**Fig. 2:**
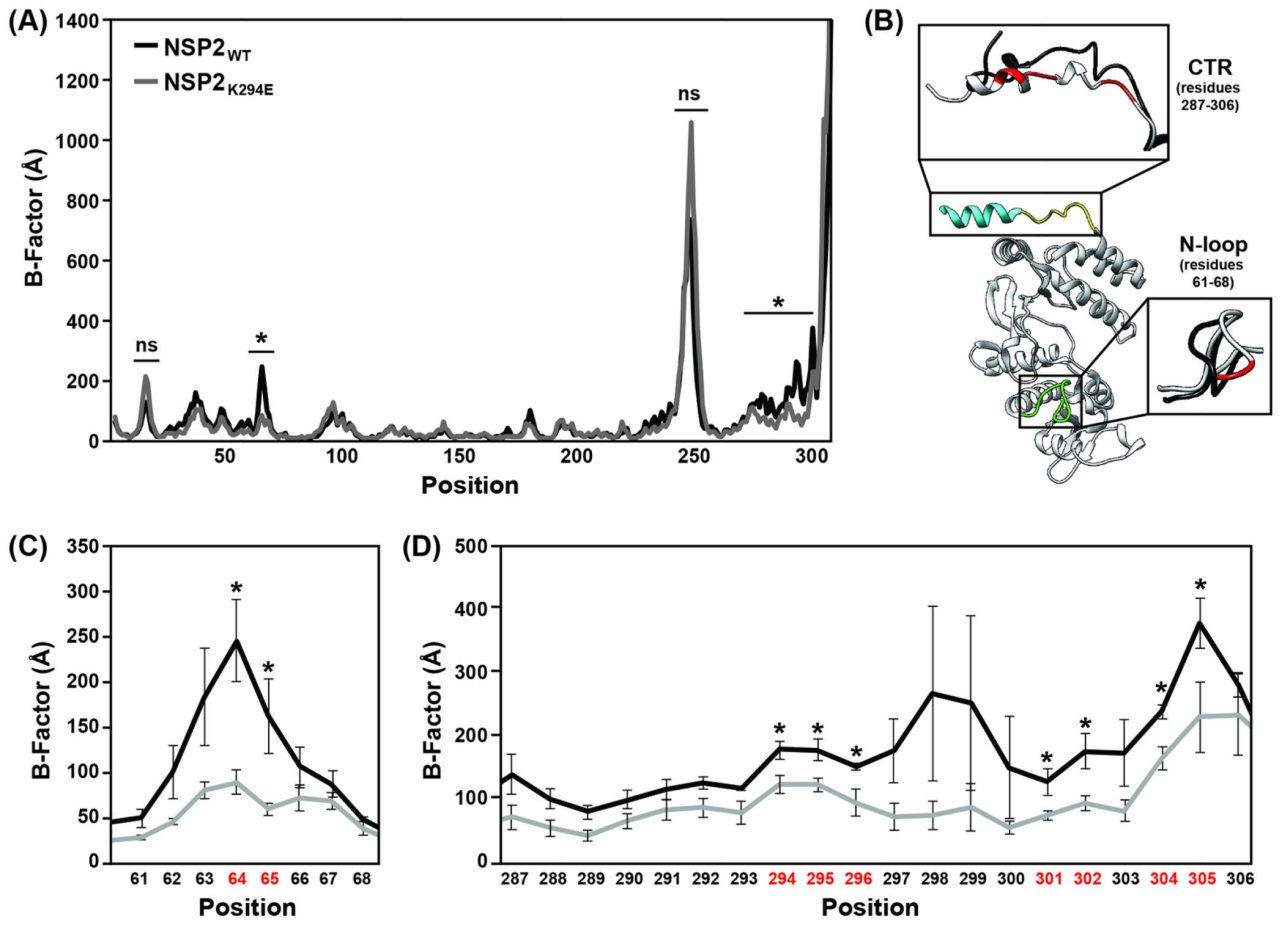
*In silico* molecular dynamics simulations of NSP2 protein structures. (A) Average B-factors are shown for either NSP2_WT_ (black line) or a molecular model of NSP2_K294E_ (grey line). Residue position numbers are shown on the x-axis. (B) Ribbon structure of the NSP2_WT_ showing regions of dynamic change. The CTR is colored as in Fig. 1, and an N-terminal loop (residues 61-68) is colored green. The dominant trajectory models of these regions are shown as insets with NSP2_WT_ in black and NSP2_K294E_ in grey. Residues with B-factors that differ between NSP2_WT_ and NSP2_K294E_ are colored red. (C) Average B-factors for residues 61-68. Colors are the same as in panel A. Error bars represent standard error from the mean following three independent simulations. Statistical significance was determined using a two-tailed T test. Asterisks (*) indicate statistically significant differences (*p*<0.05). ns=not significant. (D) Average B-factors for CTR residues 287-306. Colors are the same as in panel A. Error bars represent standard error from the mean following three independent simulations. Statistical significance was determined using a two-tailed T test. Asterisks (*) indicate statistically significant differences (*p*<0.05).

**Fig. 3:**
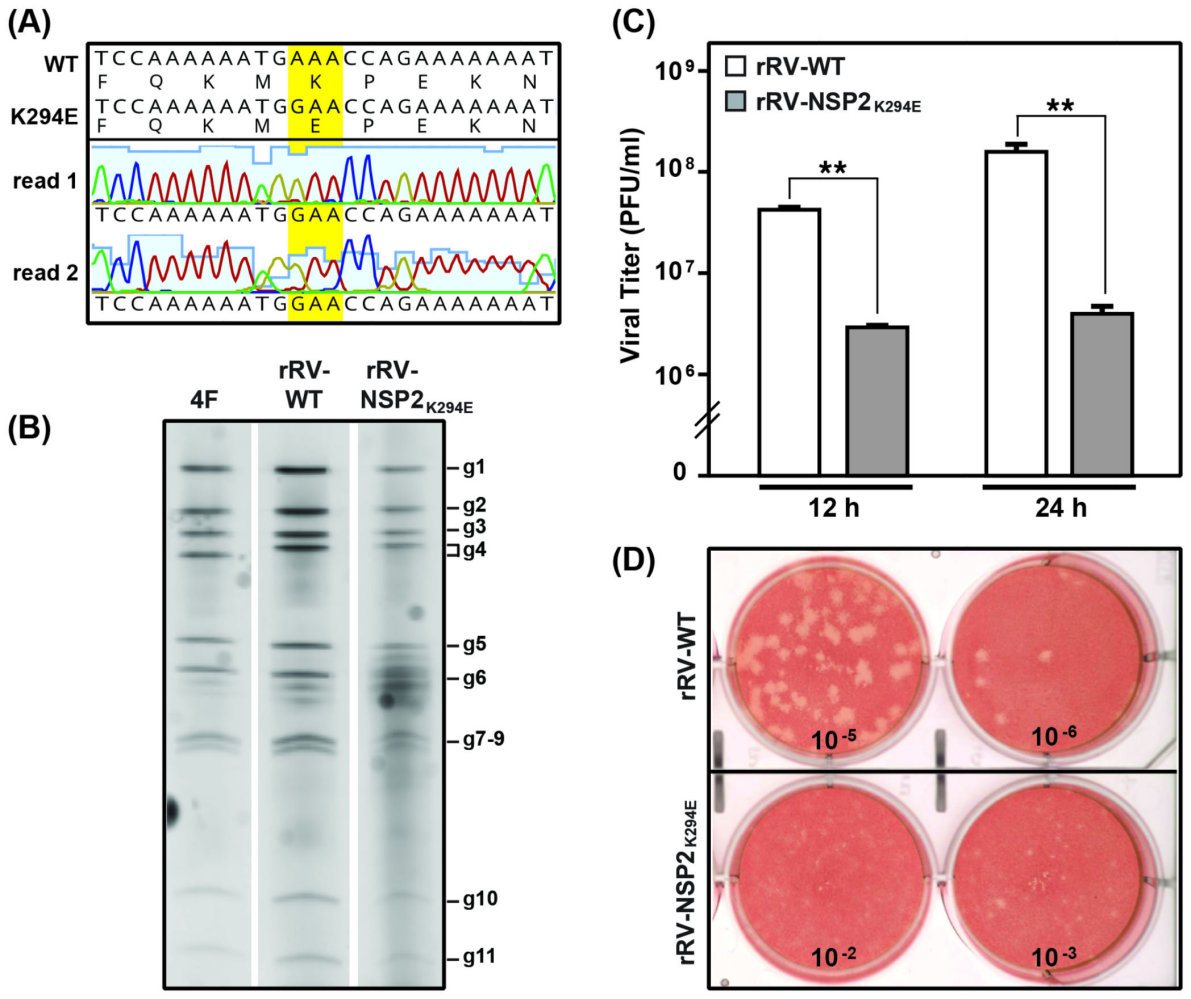
Reverse genetics rescue and replication of rRV-NSP2_K294E_. (A) Sequence analysis of the rRV-NSP2_K294E_ working stock. The NSP2-coding gene of rRV-NSP2_K294E_ was amplified by RT-PCR and subjected to Sanger sequencing. The chromatograms of two independent sequencing reactions (read 1 and read 2) are shown across the engineered lesion only. Nucleotide and amino acid sequences of the unmodified wildtype (WT) pT7-NSP2-SA11 plasmid are shown above the consensus mutant virus sequence. The engineered codon is highlighted yellow. (B) Electropherotyping. Viral dsRNA was extracted from laboratory strain SA11-4F (4F), rRV-WT, or rRV-NSP2κ294E working viral stock and visualized following SDS-PAGE and SYBER Gold staining. The positions of the 11 viral genome segments (g1-g11) are labeled to the right of the gel. (C) Single-cycle replication assay. MA104 cells were infected at an MOI of 3 PFU per cell with either rRV-WT (white) or rRV-NSP2_K294E_ (grey). Viral titers were determined by plaque assay. Error bars represent standard error from the mean. Statistical significance was determined using a two-tailed, unpaired T-test. Asterisks (**) indicate p<0.001. (C) Images of representative plaque assay wells for rRV-WT (top) or rRV-NSP2_K294E_ (bottom) at 9 d p.i. following neutral red overlay.

CTR undergoes domain-swapping interactions that link several NSP2 octamers together under crystallographic conditions (32). Given its conservation and dynamic position in the NSP2 CTR open v. closed states, K294 is expected to be a key flexibility determinant (Fig. 1B). We predicted that an aspartic acid substitution at this site (K294E) would alter flexibility of the NSP2 CTR due to the charge swap. To test this prediction, *in silico* molecular dynamics simulations were performed (Fig. 2). Specifically, the atomic structure of wildtype NSP2 (NSP2_WT_) was used to create a homology model containing the K294E change (NSP2_K294E_), and computational simulations using GROMACS v2020.4 were performed on individual monomers. Root mean square deviations of the α-carbons were used to calculate a B-factor for each residue. In this analysis, a larger B-factor indicates more flexibility at a given position and is used as a readout for conformational changes. The results of three independent trajectories revealed several regions of high dynamic flexibility in both the NSP2_WT_ and NSP2_K294E_ (Fig. 2A). However, only two regions of the protein had B-factors that differed significantly between NSP2_WT_ and NSP2_K294E_, which are suggestive of conformational changes induced by the K294E substitution (Fig. 2B, 2C, and 2D). Specifically, positions 64 and 65 of an N-terminal loop region (comprised of residues 61-68) had slightly reduced flexibility in NSP2_K294E_ as compared to NSP2_WT_ (Fig. 3B). More dramatically, the entire CTR of the NSP2_K294E_ mutant was reduced in its flexibility, with 9 positions (288, 289, 294-296, 301, 302, 304 and 305) having B-factors that differed significantly from those of NSP2_WT_ (Fig. 3C). These computational results are consistent with the notion that reduced CTR flexibility is a major structural impact of the K294E change.

### Rescue of a replication-defective mutant RV bearing the K294E NSP2 change

We next sought to determine whether we could rescue a recombinant mutant RV with an engineered K294E change (rRV-NSP2_K294E_) using the fully plasmid-based reverse genetics system for strain SA11 developed by Kanai et al. (37). This system has several benefits over the previous NSP2 helper virus-based system, as it allows for the rescue of highly replication-defective mutants (12). Site-directed mutagenesis was used to incorporate a single nucleotide substitution into the pT7-NSP2-SA11 plasmid, encoding a K294E change in NSP2. The mutant plasmid was then used in transfection reactions *in lieu* of the wildtype plasmid. Specifically, the 11 pT7 plasmids (i.e., one for each viral gene segment) were transfected into T7 polymerase-expressing baby hamster kidney (BHK-T7) cells along with support plasmids encoding an RNA capping enzyme and a small fusion protein. Viruses generated in BHK-T7 cells were amplified by infection of monkey kidney MA104 cells. Using this approach, we successfully rescued rRV-NSP2_K294E_, albeit with delayed kinetics as compared to the recombinant wildtype control virus (rRV-WT). The NSP2-coding gene was amplified by reverse transcriptase-polymerase chain reaction (RT-PCR) from purified rRV-NSP2_K294E_ RNA and sequenced across the open reading frame (ORF) to confirm the incorporation of the engineered change and the absence of second-site mutations (Fig. 3A). While we did not sequence the entire genome of rRV-NSP2_K294E_, we took care to minimally passage the viral stock to mitigate the potential for compensatory, adaptive changes. As expected, no major differences were detected in the electrophoretic migration of the 11 dsRNA genome segments for rRV-WT versus rRV-NSP2_K294E_ (Fig. 3B).

To test the impact of the introduced mutation on viral replication, MA104 cells were infected with either rRV-WT or rRV-NSP2_K294E_ at a multiplicity of infection (MOI) of 3 plaque forming units (PFU) per cell, allowing for single-cycle infections. Cells and culture media were harvested at 0, 12, and 24 h p.i., and viral titers were determined by plaque assay. The results show that the titers of rRV-NSP2_K294E_ were significantly lower (>1.2-log) than those of rRV-WT at both the 12 and 24 h timepoints (Fig. 3C). Moreover, the plaques formed by rRV-NSP2_K294E_ were much smaller than those of rRV-WT (Fig. 3D). These results demonstrate that rRV-NSP2_K294E_ has a significant replication defect and underscore the importance of NSP2 CTR flexibility for optimal RV replication.

### NSP2_K294E_ exhibits robust NTPase activity

**Fig. 4:**
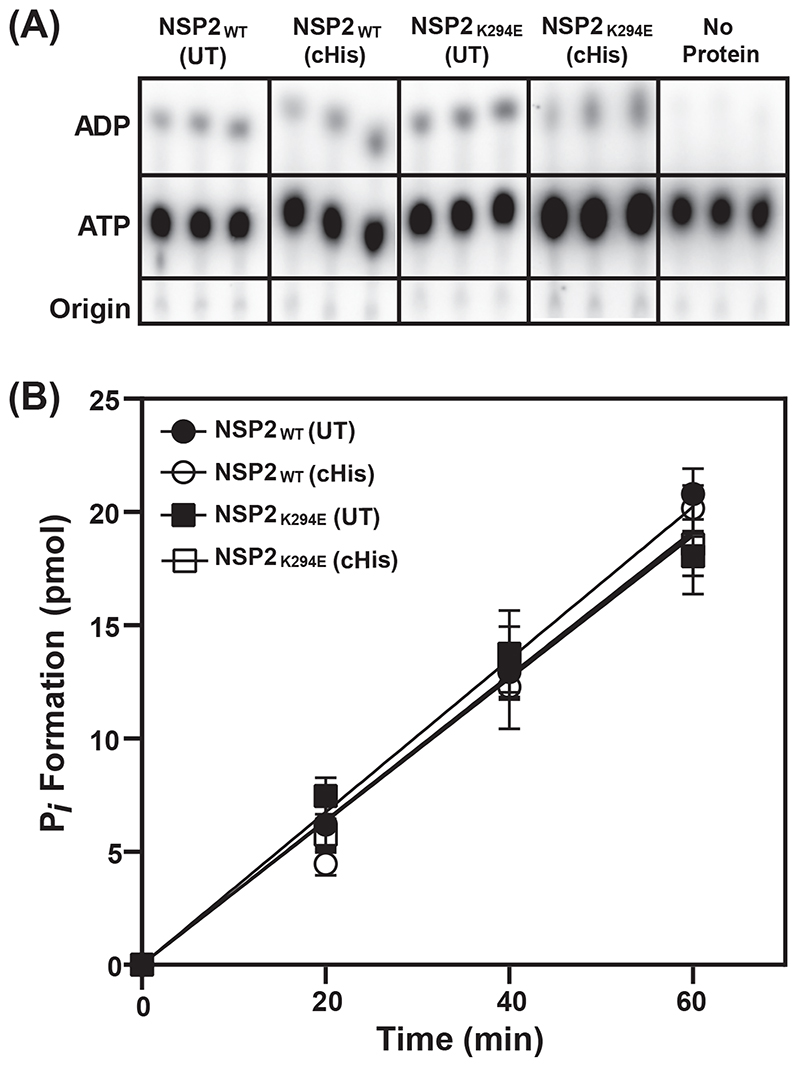
*In vitro* NTPase activity of NSP2_WT_ and NSP2_K294E_ proteins. (A) Representative TLC plate images showing hydrolysis of P*i* from [α^32^P]-ATP to produce [α ^32^P]-ADP in the presence of various cHis tagged or untagged (UT) NSP2 proteins. No protein was added in the negative control reactions. (B) Quantification of ATP hydrolysis and release of P*i* by various NSP2 proteins as described in Material and Methods. Error bars represent standard error from the mean.

Having observed the replication defect of rRV-NSP2_K294E_, we next wondered whether the K294E change impacted the capacity of NSP2 to function as an octameric enzyme. To test this, recombinant C-terminal hexahistidine (cHis) tagged NSP2_K294E_ and NSP2_WT_ control proteins were expressed in *Escherichia (E.) coli* and purified using Co^2+^affinity chromatography. The cHis tags were either maintained or thrombin-cleaved to create untagged (UT) NSP2 proteins. To test NTPase activity, the NSP2 proteins were incubated with [α^32^P]-ATP for 0, 20, 40 and 60 min. The [α^32^P]-ADP reaction products of NTPase activity were separated from input [α^32^P]-ATP using thin layer chromatography (Fig. 4A). Input and product spots were quantified using a phosphorimager (Fig. 4B). The results showed that NSP2_K294E_ (UT) and NSP2_K294E_ (cHis) proteins converted [α^32^P]-ATP to [α^32^P]-ADP at rates that were indistinguishable from each other and from those of the control proteins NSP2_WT_ (UT) and NSP2_WT_ (cHis) (Fig. 4B). These results suggest that the K294E change does not influence the enzymatic activity of NSP2 *in vitro* (39). Thus, the replication defect of rRV-NSP2_K294E_ is unlikely to be caused by changes in the NTPase (or RTPase) activity of NSP2. These results further suggest that the cHis tag does not alter NSP2 function. Such a notion is also validated by Bravo et al., who reported the reverse genetic rescue of a highly replication-competent rRV expressing a cHis-tagged NSP2 protein (30).

### Numerous, smaller viroplasms in rRV-NSP2_K294E_-infected cells

**Fig. 5:**
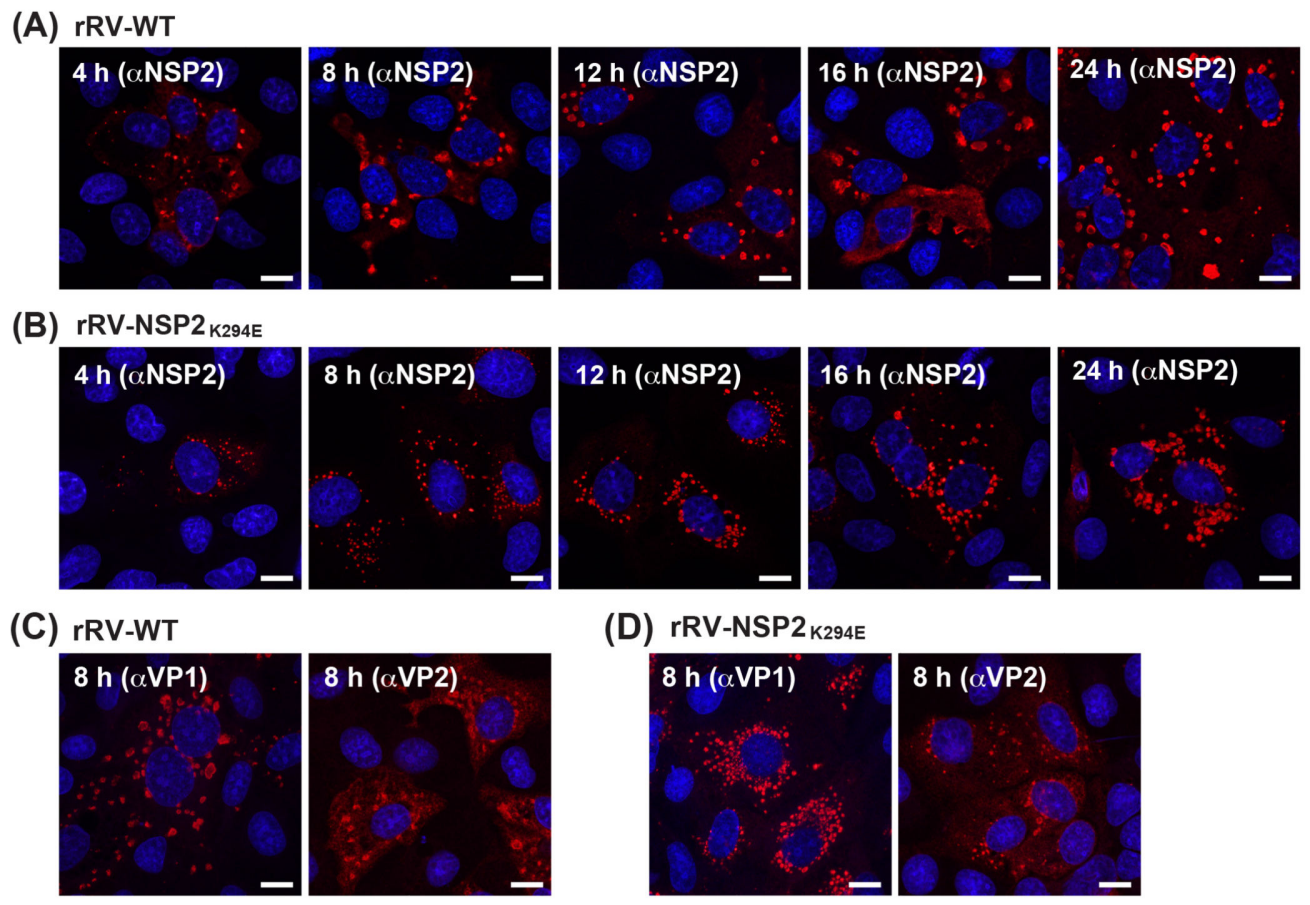
Confocal imaging of viroplasms in rRV-WT- or rRV-NSP2_K294E_-infected cells. MA104 cells on glass coverslips were infected with rRV-WT or rRV-NSP2_K294E_ at an MOI of 3 PFU per cell for 4, 8, 12, 16 or 24 h. (A and B) Cells were fixed with methanol and stained using αNSP2 and an Alexa-546 conjugated secondary antibody. Nuclei were stained using Hoechst. Confocal microscopy was used to determine the localization of NSP2 (561-nm; red) and nuclei (405-nm; blue). Scale bar indicates 10 μm. (C and D) Cells were fixed with methanol and stained using αVP1 or αVP2 and an Alexa-546 conjugated secondary antibody. Nuclei were stained using Hoechst. Confocal microscopy was used to determine the localization of VP1 or VP2 (561-nm; red) and nuclei (405-nm; blue). Scale bar indicates 10 μm.

Because we found no differences in NSP2_K294E_ NTPase activity, we next wanted to visualize NSP2 localization in the context of rRV-NSP2_K294E_ infection to see if the engineered change impacted viroplasms. To do this, MA104 cells on glass coverslips were either mock infected or infected at a MOI of 3 PFU per cell with either rRV-WT or rRV-NSP2_K294E_ for 4, 8, 12, 16, or 24 h. Coverslips were fixed with methanol, immunostained using αNSP2 and imaged by confocal microscopy (Fig. 5A-B). We observed numerous, smaller viroplasms in rRV-NSP2_K294E_-infected cells at each timepoint as compared to those in rRV-WT-infected cells (Fig. 5A-B). While the rRV-WT viroplasms appeared larger as infection proceeded, few large-sized viroplasms were observed for rRV-NSP2_K294E_ even at the 24 h timepoint (Fig. 5B). Similar results were obtained when cells were stained with αVP1 or αVP2 antiserum, suggesting that the small viroplasm phenotype of rRV-NSP2_K294E_ was not simply due to differences in αNSP2 epitope recognition (Fig. 5C-D).

**Fig. 6:**
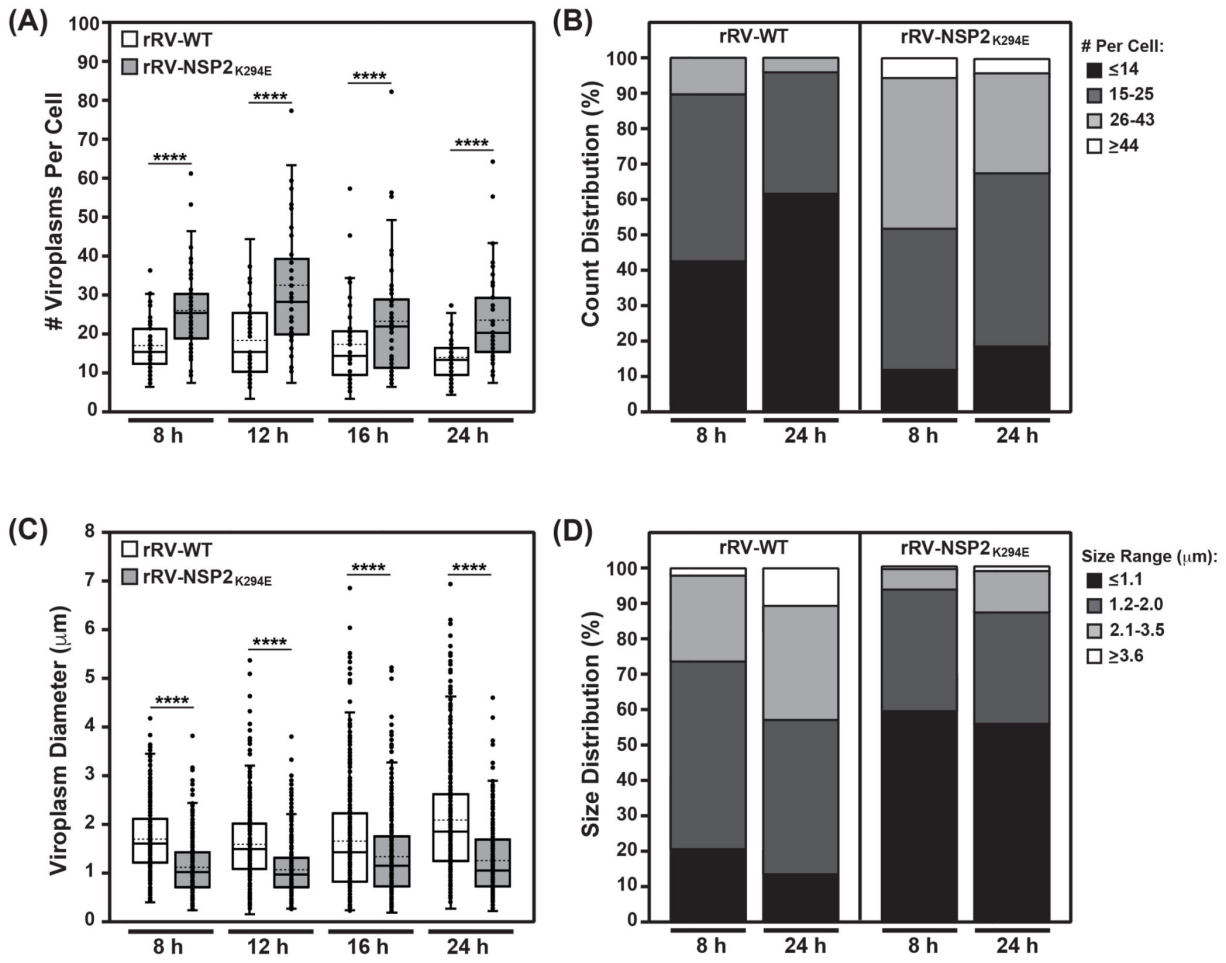
Quantification of viroplasm number and size. (A) The number of viroplasms per cell for rRV-WT (white) or rRV-NSP2_K294E_ (grey) was quantified in blinded αNSP2-stained micrographs at each time point. The mean is shown as a dashed line and the median is shown as a solid line in the box and whiskers plot. Statistical significance was determined using the negative binomial model. Asterisks (****) indicate p<0.0001. (B) The data for viroplasm numbers at the 8 and 24 h timepoints were binned using a custom R script. Each bin represents the percentage of cells with the viroplasm numbers according to the legend. (C) The diameter of viroplasms for rRV-WT (white) or rRV-NSP2_K294E_ (grey) was quantified in blinded αNSP2-stained micrographs at each time point. The mean is shown as a dashed line and the median is shown as a solid line in the box and whiskers plot. Statistical significance was determined using the Box-cox transformation followed by a linear regression. Asterisks (****) indicate p<0.0001. (D) The data for viroplasm diameters at the 8 and 24 h timepoints were binned using a custom R script. Each bin represents the percentage of cells containing viroplasms with the size range indicated in the legend.

Quantification of the blinded confocal micrographs from the αNSP2 staining revealed heterogeneity in the number of viroplasms per cell at each timepoint for both viruses (Fig. 6A-B). Still, the number of viroplasms per cell were significantly higher for rRV-NSP2_K294E_ as compared to rRV-WT at all time points tested (Fig. 6A). This increased viroplasm number for the mutant virus was even more apparent after binning the data from the 8 and 24 h timepoints based on cell distribution (Fig. 6B). Specifically, at 8 h p.i., only 10% of rRV-WT-infected cells contained ≤26 viroplasms, while 48% rRV-NSP2_K294E_-infected cells contained ≤26 viroplasms (Fig. 6B). Even at the late timepoint of 24 h p.i., 62% of rRV-WT-infected cells contained <14 viroplasms, while only 20% the of rRV-NSP2_K294E_-infected cells contained <14 viroplasms (Fig. 6B).

A high degree of heterogeneity was also observed when measuring the sizes of viroplasms in blinded confocal micrographs from the αNSP2 staining (Fig. 6C-D). Still, the viroplasm sizes were significantly lower for rRV-NSP2_K294E_ as compared with rRV-WT at each timepoint (Fig. 6C). Binning the data from the 8 and 24 h timepoints further supported the notion that viroplasms were smaller for rRV-NSP2_K294E_ (Fig. 6D). In particular, at 8 h p.i., only 22% of rRV-WT-infected were ≤1.1 μm in diameter, whereas 60% of rRV-NSP2_K294E_-infected cells contained these very tiny inclusions (Fig. 6D). Even by 24 h p.i., when 43% of rRV-WT viroplasms were ≥2.1 μm in diameter, only 12% of viroplasms reached this larger size for the rRV-NSP2_K294E_ mutant (Fig. 6D).

**Fig. 7:**
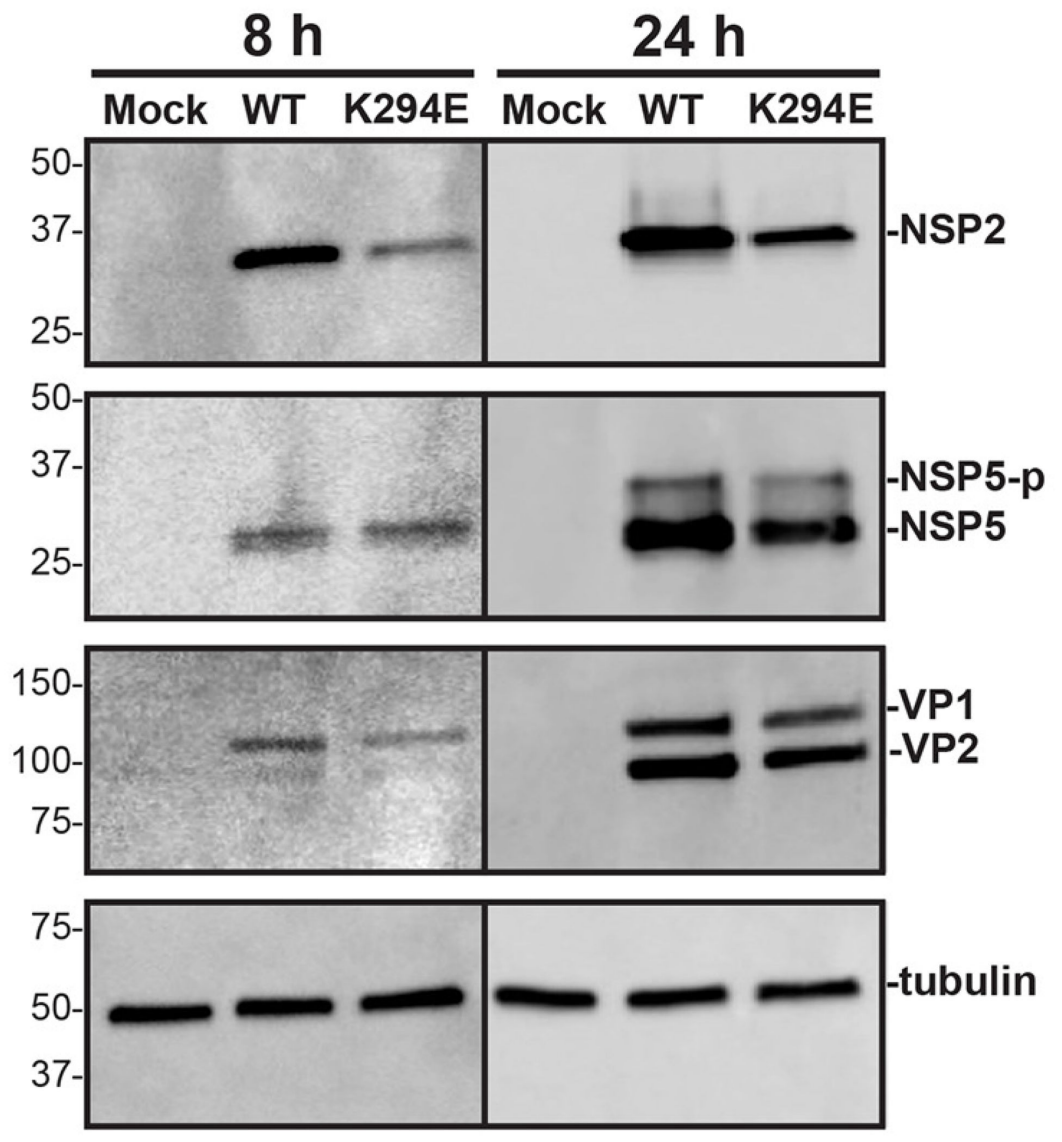
Western blot of NSP2 and NSP5. MA104 cells were either mock infected or infected with rRV-WT or rRV-NSP2_K294E_ at an MOI of 3 PFU per cell for 8 or 24 h. Lysates were subjected to western blot using αNSP2, αNSP5 aVP1/VP2, or αtubulin as a loading control. Secondary antibodies conjugated to HRP were used for detection. Molecular weight markers (in kDa) are shown to the left of the blots, and the locations of protein of interest are labeled on the right. Hyper-phosphorylated NSP5 (NSP5-p).

To test whether viral proteins levels were severely altered for rRV-NSP2_K294E_, mock or infected MA104 cell lysates were analyzed by western blot using αNSP2, αNSP5, aVP1/VP2, or atubulin as a loading control (Fig. 7). The results showed slightly decreased levels of viral proteins at both timepoints for rRV-NSP2_K294E_ as compared to rRV-WT (Fig. 7). Because rRV-NSP2_K294E_ has a significant replication defect (Fig. 2), it is not possible to uncouple whether reduced protein levels are a cause or consequence of the small numerous viroplasm phenotype. Still, the phosphorylated isoforms of NSP5 detected with our αNSP5 antisera appeared to be indistinguishable between rRV-NSP2_K294E_ and rRV-WT at 24 h p.i., which is a timepoint when the mutant virus has significantly more numerous and smaller viroplasms (Fig. 6A and 6C). These results suggest that the NSP5 phosphorylation status alone does not explain the viroplasm phenotype of rRV-NSP2_K294E_.

### Delayed fusion of viroplasms in rRV-NSP2_K294E_-infected cells

**Fig. 8:**
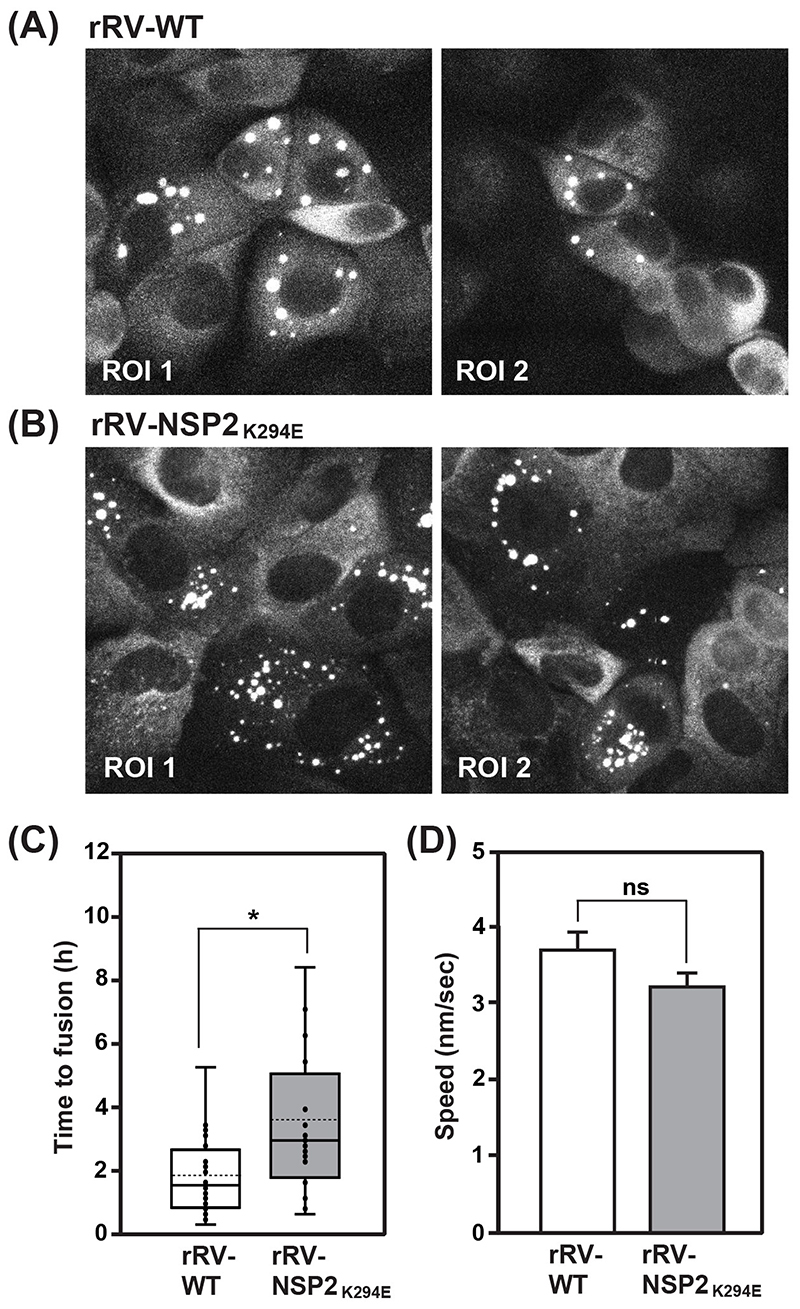
Live-cell imaging of viroplasms in NSP2wτ:mCherry expressing MA104 cells. MA104 cells stably expressing NSP2_WT_ :mCherry were infected with rRV-WT (A) or rRV-NSP2_K294E_ (B) at an MOI of 3 PFU per cell for 16 h. Cells were imaged every 10 min using confocal microscopy. Representative still images of two regions of interest (ROI 1 and ROI 2) are shown at 16 h p.i. Viroplasms were tracked as described in the Materials and Methods to measure the time to fusion and average speed. (C) Time to fusion. Numbers are shown in h. The mean is shown as a dashed line and the median is shown as a solid line in the box and whiskers plot. A total of 20 individual fusion events were analyzed for each virus. Statistical significance was determined using a two-tailed, unpaired T-test. The asterisk (*) indicates p<0.05. (D) Viroplasm speed. Numbers are shown in nm/sec. A total of 90 viroplasms were tracked per virus. Statistical significance was determined using a two-tailed, unpaired T-test. Error bars represent the standard error from the mean. ns=not significant.

**Fig. 9:**
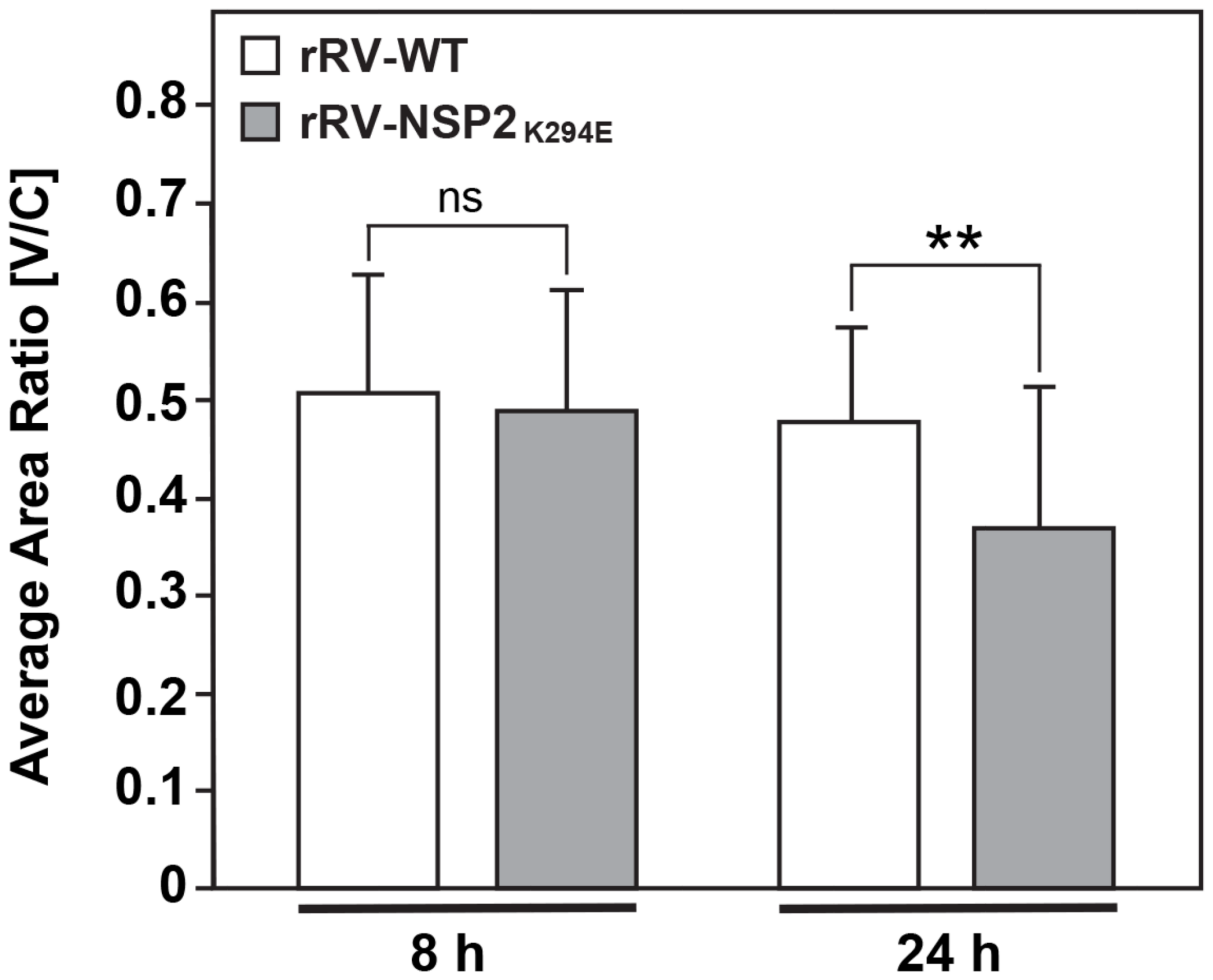
Perinuclear localization of viroplasms. MA104 cells on glass coverslips were infected with rRV-WT or rRV-NSP2_K294E_ at an MOI of 3 PFU per cell for 8 or 24 h. Cells were fixed with methanol and stained using αNSP2 and an Alexa-546 conjugated secondary antibody. Nuclei were stained using Hoechst. Confocal microscopy was used to determine the localization of NSP2 (561-nm; red) and nuclei (405-nm; blue). Perinuclear condensation [V/C ratio] was determined as described in the Methods. Statistical significance was determined by a two-tailed, unpaired T-test. ns=not significant. Asterisks (**) indicate p<0.01.

The fixed cell immunofluorescence data showing numerous, small viroplasms for rRV-NSP2_K294E_ led us to consider whether the K294E change in the NSP2 CTR may have impacted viroplasm fusion events (i.e., the merging of small viroplasms into larger condensates). To test this, we turned to live-cell confocal imaging. Specifically, we employed an MA104 cell line that stably expresses low levels of wildtype NSP2 with the red fluorescent protein mCherry fused to its C-terminus (NSP2wτ:mCherry) (Fig. 8). Previous studies have shown that this NSP2wτ:mCherry fusion protein is diffusely distributed in mock-infected cells, but that >90% of the protein relocalizes to viroplasms upon rotavirus infection (16). Thus, the C-terminal mCherry tag does not abrogate the capacity of ectopic NSP2 to become enriched into the RV factories. Here, the NSP2wτ:mCherry-expressing MA104 cells were infected with either rRV-WT or rRV-NSP2_K294E_ or at a MOI of 3 PFU per cell and then imaged every 10 min for 16 h using confocal microscopy and the 561-nm laser line. Individual viroplasms were then tracked using image analysis software to determine their fusion rates and speed. Like the results of fixed-cell immunofluorescence assays, the live-cell imaging experiments revealed numerous, smaller viroplasms in rRV-NSP2_K294E_-infected cells versus rRV-WT-infected cells (Fig. 8A and 8B). This observation suggested that the rRV-NSP2_K294E_ phenotype was not complemented *in trans* by the low levels of NSP2wτ:mCherry protein. Analysis of viroplasm fusion rates from the live cell imaging data showed that those in rRV-NSP2_K294E_ infected cells took significantly longer to fuse than did those in control virus-infected cells (Fig. 8C). While the average speed for rRV-NSP2_K294E_ viroplasms was slightly slower than those of rRV-WT, though the results were not statistically significant (Fig. 8D). A previous report indicated that RV factories migrate towards the nucleus as infection proceeds because of their movement along microtubules (13). Image analyses of our fixed cell immunofluorescence data showed no differences in the perinuclear distribution of rRV-NSP2_K294E_ or rRV-WT viroplasms at 8 h p.i. (Fig. 9). However, at 24 h p.i., rRV-NSP2_K294E_ viroplasms were slightly but significantly closer to the nucleus than those of rRV-WT (Fig. 9). This result suggests that the slower speed and reduced time-to-fuse for the mutant are unlikely to be caused by defects in NSP2-microtubule interactions. The numerous, smaller viroplasms for rRV-NSP2_K294E_ combined with their decreased fusion may reflect a reduced capacity of NSP2_K294E_ to undergo efficient LLPS alongside NSP5.

### NSP2 CTR mutants have defects in viroplasm-like structure (VLS) formation

**Fig. 10:**
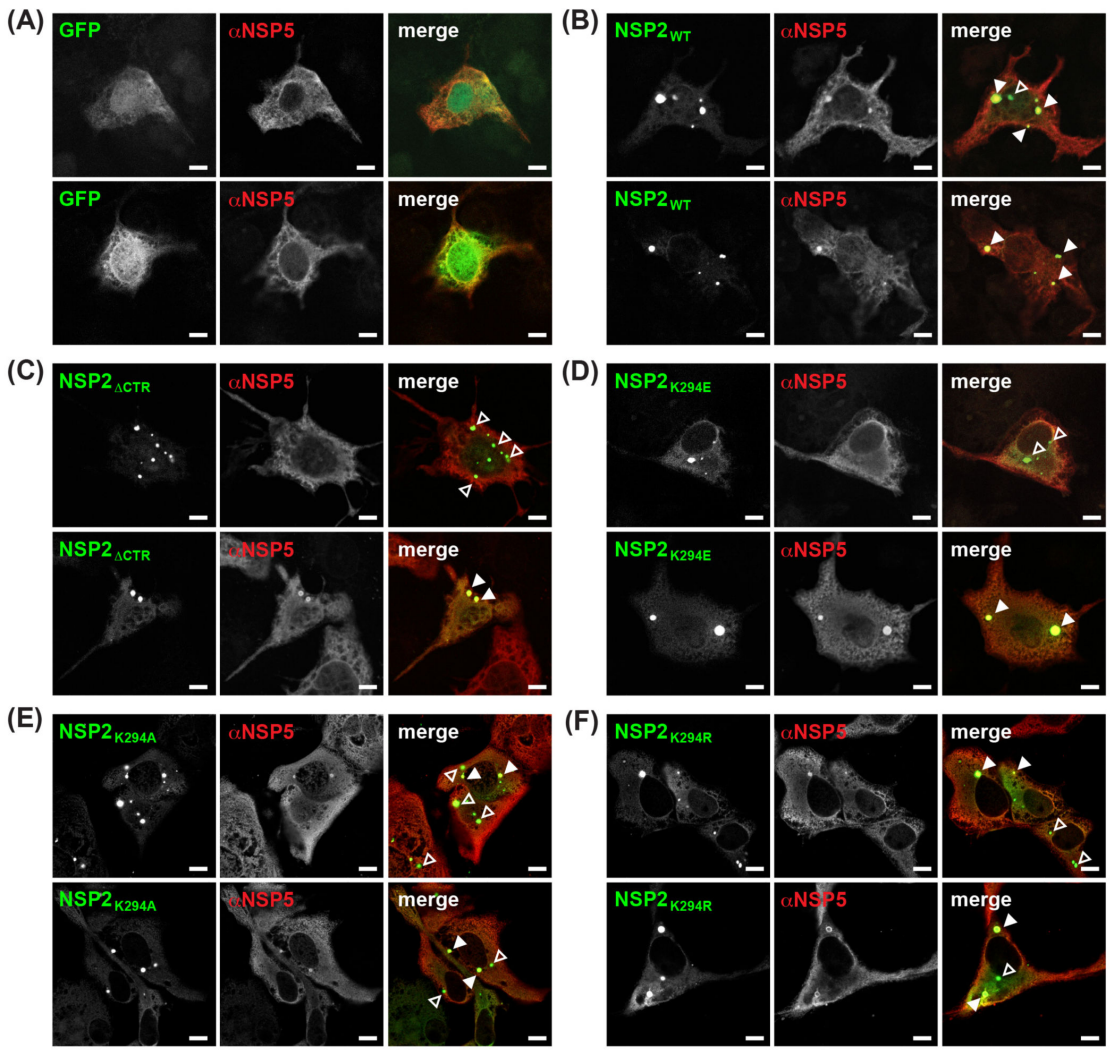
VLS formation by NSP2 CTR mutants. Cos-7 cells on glass coverslips were transfected with plasmids expressing untagged NSP5 and either GFP only (A) or the following NSP2-GFP fusion proteins: NSP2_WT_(B), NSP2_ΔCTR_ (C), NSP2κ294E (D), NSP2κ294A (E), or NSP2κ294R (F). At 48 h post-transfection, cells were fixed with methanol and stained using αNSP5 and an Alexa-546 conjugated secondary antibody. Confocal microscopy was used to determine the localization of GFP signal (488-nm; green) relative to αNSP5 staining (561-nm; red). Co-localization of green and red is shown as yellow in the merged images. The location of punctate VLSs formed of NSP2 and NSP5 are indicated with closed arrowheads, while aggregates of NSP2 fusion proteins are indicated with open arrowheads. Scale bar indicates μm.

While not recapitulating all aspects of *bona fide* viral factories, VLS that result from the co-expression of NSP2 and NSP5 in cells (in the absence of RV infection) are widely used as a readout for viroplasm nucleation and LLPS inclusion formation (16, 20–22). In this assay, NSP2 can be tagged with enhanced green fluorescent protein (GFP) at its C-terminus without affecting (i) its capacity to form VLS alongside untagged NSP5 or (ii) its capacity to localize *in trans* to viroplasms during infection (21). A previous study reported that an NSP2 mutant lacking CTR residues 293-317 (NSP2_ΔCTR_) did not form VLS when co-expressed with NSP5 in MA104 cells (36). Therefore, we sought to test the capacity of NSP2_K294E_ to form VLS relative to both NSP2_WT_ (positive control) and to NSP2_ΔCTR_ (negative control). To do this, monkey kidney Cos-7 cells on glass coverslips were co-transfected with plasmids expressing untagged NSP5 and either GFP only (i.e., empty vector) or one of the NSP2-GFP fusion proteins. At 48 h post-transfection, the cells were fixed with methanol, immunostained using αNSP5 guinea pig polyclonal antiserum and an Alexa-546 fluorescently conjugated secondary antibody, and then imaged by confocal microscopy using 488-nm and 561-nm laser lines (Fig. 10). We observed that all the NSP2-GFP fusion proteins, including NSP2_WT_, formed punctate aggregates when highly expressed in Cos-7 cells (data not shown). Thus, only foci that were enriched for both NSP2 and NSP5 (i.e., observed in both the 488-nm and 561-nm channels) were scored as VLS.

As expected, no VLS formed in cells co-expressing NSP5 and GFP only (i.e., no NSP2); diffuse fluorescence was instead seen in both the 488-nm and 561-nm channels (Fig. 10A). In contrast, VLSsconsistently formed in cells co-expressing NSP5 and NSP2_WT_ (Fig. 10B, closed arrowheads). To our surprise, VLS were also occasionally observed in cells co-expressing NSP5 and the negative control NSP2_ΔCTR_, suggesting that while the CTR is not essential for inclusion formation, it supports this process (Fig. 10C, closed arrowheads). Indeed, NSP2_ΔCTR_ more often accumulated in non-VLS aggregates (Fig. 10C, open arrowheads) or it was diffuse in its distribution rather than localizing to NSP5-rich VLS inclusions, consistent with a previous study (36). Interestingly, the phenotype of NSP2_K294E_ was similar to that of NSP2_ΔCTR_; this mutant co-localized with NSP5 in VLS but only in some cells (Fig. 10C, closed arrowheads). Like NSP2_ΔCTR_, NSP2_K294E_ was often seen localized to non-VLS aggregates (Fig. 10D, open arrowheads), or it was diffusely distributed in the cytosol (data not shown). To probe whether the chemistry of position 294 impacted VLS formation by NSP2 constructs containing either a lysine to alanine (positive to negative charge change) or a lysine to arginine (positive to positive) residue change at this site were tested (i.e., NSP2_K294A_ or NSP2_K294R_, respectively) (Fig. 10D and 10F). Similar phenotypes were observed for these variants, with NSP2_K294A_ or NSP2_K294R_ forming VLS in some cells, while aggregating in others (Fig. 10D and 10F).

We next quantified the number of cells with VLS in blinded confocal micrographs and compared the efficiency of the NSP2 CTR mutants to that of NSP2_WT_, which was set to 100% (data not shown). The quantification results were consistent with our observations, revealing that NPS2ΔCTR formed VLS only 20% (± 1.7% standard error (SE)) of the time. However, the point mutants NSP2_K294E_, NSP2_K294R_, and NSP2_K294A_ mutants were found to be better than NPS2δCTR, forming VLS 45% (± 2.9% SE), 46% (±0.09% SE), and 79% (± 2.7% SE) of the time. Together, these results suggest that mutation or deletion of the NSP2 CTR reduces its capacity to form factory-like condensates alongside NSP5 in cells, underscoring the importance of this flexible region in RV factory formation. The observation that NSP2_K294E_ and NSP2_K294R_ have similar phenotypes in this VLS assay suggests that a lysine residue at position 294 is important and that a positively-charged arginine does not substitute.

### NSP2_K294E_ forms smaller LLPS condensates with NSP5 *in vitro*

**Fig. 11:**
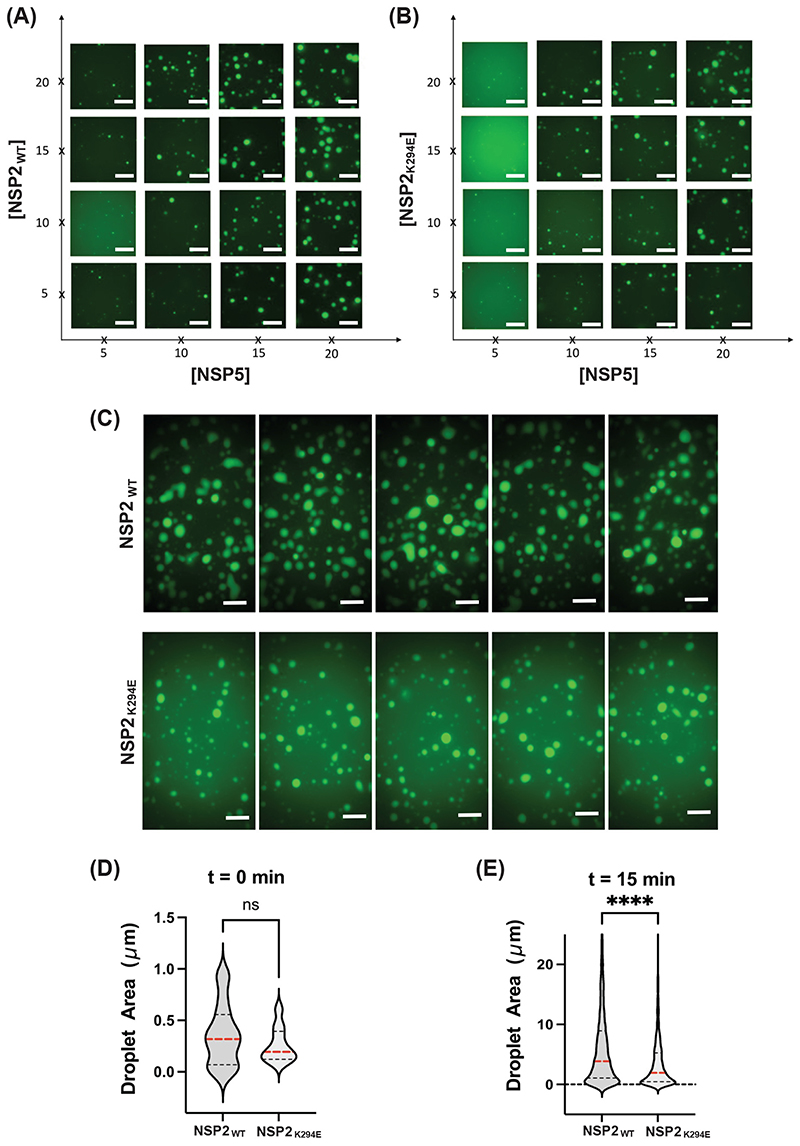
*In vitro* LLPS droplet formation. Phase diagrams generated through fluorescent imaging of recombinant unlabeled NSP5 mixed with 488Atto-labeled NSP2_WT_ (A) or NSP2K249E (B). NSP2 and NSP5 protein samples were mixed at varying concentrations (5-20 μM) as shown on both axes. Droplets formed upon the addition of NSP5 and were imaged following a 5 min incubation. Scale bar=10 μm. Droplet formation assays and sizing of NSP5/NSP2 condensates. 15 μM of the recombinant 488Atto-labeled NSP2_WT_ or the NSP2_K294E_ was mixed with 15 μM of NSP5. Droplet formation was imaged directly after the proteins were mixed (not shown), and 15 min following the incubation upon their formation to allow them to fuse (C). Five microfluidic wells are shown for each protein. Scale bar=10 μm. (D) Droplet sizes were quantified immediately after their formation and (E) 15 min after their formation. Droplets were detected and counted as described in Materials and Methods. ns=not significant for t = 0 min. Statistical significance was determined using a two-tailed, unpaired T-test. The asterisks (**** represent *p<* 0.0001. Red lines denote the mean, black lines show the quartiles.

While the VLS assay provides a binary read-out for inclusion formation in an intact cell, it is not amenable to understanding the mechanistic details of how NSP2 and NSP5 phase separate to form RV factories. To test whether NSP2K294 has defects in its capacity to undergo LLPS, we next employed an *in vitro* assay that was recently developed by Geiger et al. (16). In this assay, purified, recombinant cHis-tagged NSP2 is fluorescently labeled (Atto488) and incubated with purified, recombinant N-terminally StrepII-tagged NSP5 protein. At physiologically relevant, low μM concentrations, the NSP2 and NSP5 proteins form condensates *in vitro,* which can be visualized as droplets and measured microscopically. Here, we incubated various concentrations (5-20 μM) of Atto488-labeled NSP2_WT_ or NSP2_K294E_ proteins with various concentrations (5-20 μM) of unlabeled NSP5 for 5 min and then imaged the droplets to generate phase diagrams (Fig. 11A-B). The results show that while NSP2_K294E_ formed *in vitro* LLPS droplets with NSP5, they were smaller and less numerous than those formed by NSP2_WT_ at the same protein concentrations (Fig. 11A-B). Moreover, at sub-saturating levels of NSP5 (15 μM), ~2-3X more NSP2_K294E_ protein (i.e., ~15-20 μM) was required to form the same number/size of droplets as NSP2_WT_ (i.e, ~5-10 μM) (Fig. 11A-B).

To quantify the size of these droplets, we mixed 15 mM of the 488-labeled NSP2_WT_ or NSP2_K294E_ protein with 15 μM of unlabeled NSP5. Droplets were imaged directly after the proteins were mixed (0 min) or after 15 min of incubation, which is the amount of time it takes for smaller droplets to fuse together to form larger condensates (Fig. 11C). Quantification of droplet area showed that those formed by NSP2_K294E_ at 0 min were smaller on average than those of NSP2_WT_, but the results were not statistically significant (Fig. 11D). However, after the 15 min incubation period, the NSP2_K294E_ droplets were found to be significantly smaller than those of NSP2_WT_ (Fig. 11E). These results demonstrate that the K294E change in the NSP2 CTR diminished the capacity of the NSP2 to undergo phase separation with NSP5, providing a mechanistic explanation for the viroplasm phenotype of rRV-NSP2_K294E_.

## Discussion

Many viruses induce the formation of viral factories within the infected host cell, which serve as the sites for viral RNA synthesis, viral protein synthesis, and virion particle assembly (10). These factories are membraneless structures that have biophysical properties of biomolecular condensates, and emerging evidence suggests that some are nucleated via the process of LLPS (40, 41). Hallmarks of LLPS are the formation of spherical droplets through phase separation of proteins and/or nucleic acid (42). The droplets are dynamic and fuse together over time, allowing the virus to accumulate and consolidate needed resources (42). Indeed, it has been shown that RV viroplasms are biomolecular condensates formed in part by the phase separation of non-structural proteins NSP2 and NSP5 (16). While it is likely that viral RNA and additional proteins (e.g., VP1-VP3 and VP6) are involved in viroplasm biogenesis and maturation *in vivo,* NSP2 and NSP5 alone are sufficient for the formation of factory-like VLSs in cells and for LLPS droplet formation *in vitro* (16). Still, many unanswered questions remain about the regions and residues of NSP2 that are critical for LLPS and for RV factory formation.

The NSP2 CTR (residues 291-317) is flexible and can adopt two different conformations (open v. closed) in the octamer under crystallographic conditions (32). The open conformation participates in domain-swapping interactions that link neighboring NSP2 octamers together under crystallographic conditions. Criglar et al. showed that deletion of the CTR hampers VLS formation in NSP5 co-expressing cells (36). Because the NSP5-binding site on NSP2 was mapped to the exterior grooves of the octamer, distinct from the CTR, it was predicted that the VLS-negative phenotype of the CTR-deletion mutant reflects issues with inter-octamer interactions rather than defective NSP5 interactions (29, 36, 43). Flexibility of the CTR, and therefore perhaps domain swapping, is dictated in large part by a highly conserved lysine at position 294. Our *in silico* molecular dynamics simulations suggest that a K294E change caused a significant reduction in flexibility at CTR positions 288, 289, 294-296, 301, 302, 304, and 305. Subtle dynamic differences were also seen at positions 64 and 65 in a flexible N-terminal loop region, which could have additional albeit unknown effects on NSP2_K294E_ function. Still, the major impact of the K294E change on CTR flexibility was interesting to us. We posited that the K294E change might negatively impact RV factory formation and thus reduce viral replication. The data presented here is consistent with this notion, as we found that the NSP2_K294E_ protein has a reduced capacity to form VLSs in NSP5 co-expressing cells and defects in LLPS droplet formation *in vitro*. Moreover, a mutant virus with the engineered K294E change (rRV-NSP2_K294E_) is severely reduced in its replication. Interestingly, the rRV-NSP2_K294E_ virus formed smaller and more numerous viroplasms throughout the cytoplasm of the infected cells. Live cell imaging experiments suggested that the K294E change caused issues with viroplasm fusion events. Still, whether the viroplasm phenotype of rRV-NSP2_K294E_ (and even the LLPS defect of recombinant NSP2_K294E_) is really the result of reduced inter-octamer interactions remains to be experimentally determined. Ongoing and future experiments in our lab seek to test the capacity of recombinant NSP2_K294E_ octamers to interact using size exclusion chromatography and multi-angle light scattering (SEC-MALS) and X-ray crystallography. Moreover, because the NSP2 CTR is also important for NSP2-RNA interactions, it is possible that the K294E change also impacted this facet of virus biology. Specifically, it was recently reported that deletion of the NSP2 CTR causes the protein to be kinetically trapped in an RNA-bound state *in vitro,* thereby abrogating its RNA “matchmaking” activity (30). Nevertheless, VLS formation was not diminished by a mutant NSP2 containing alanine mutations in key RNA chaperone residues (D306, D310, and E311) (30). Thus, we think that the critical function of the NSP2 CTR during viroplasm formation is likely to be distinct from its roles in RNA interactions/genome packaging. Nevertheless, future experiments would be required to formally test whether the NSP2_K294E_ protein has RNA interaction defects.

It is also important to note that while VLS formation and LLPS droplet formation are simple *in vitro* systems to study determinants of RV factory formation, they do not recapitulate several key steps of viroplasm biogenesis known to occur in the infected cells. In particular, RV factory formation *in vivo* requires an ordered phosphorylation cascade involving both NSP2 and NSP5 (12). NSP2 exists in two distinct forms in the infected cell: (i) a disperse, cytosolic form (dNSP2) that can interact with hypo-phosphorylated NSP5 and acetylated tubulin and (ii) a viroplasm-associated form (vNSP2) that is phosphorylated at CTR residue S313 by casein kinase 1 alpha, can interact with hyper-phosphorylated NSP5, and can form lattice-like NSP2 structures (12, 36). A mutant RV bearing a phosphomimetic S313D change, which would be presumed to mimic vNSP2, exhibited defects in early viroplasm formation and could not interact with lipid droplets, a cellular factor implicated in factory formation (12, 35). This result suggests that phosphorylation (and likely the dNSP2-to-vNSP2 transition) needs to be tightly regulated during infection. At this time, we cannot exclude the possibility that rRV-NSP2_K294E_ altered this delicate NSP2 phosphorylation cascade and/or interactions with host component. In the future, it would be interesting to analyze the distribution of dNSP2 and vNSP2 in rRV-NSP2_K294E_ infected cells. We found no differences in the migration pattern of NSP5 in rRV-WT versus rRV-NSP2_K294E_ infected cells at 24 h p.i., suggesting that NSP5 phosphorylation does not explain the phenotype of the rRV-NSP2_K294E_ mutant. NSP2 is also known to participate in direct or indirect interactions with several other viral and cellular proteins (e.g., VP1, VP2, VP3, NSP6, lipid droplet proteins, etc.) (17–19). Understanding the impact of the K294E change on the multi-faceted protein-protein interactions of NSP2 is an important area of future research. Still, the results presented here move us one step closer towards understanding the regions and residues of NSP2 that support RV factory formation via the process of LLPS. Such knowledge about NSP2 may broadly inform an understanding of how biomolecular condensates are nucleated in the context of other viral infections and/or in under conditions of normal cellular physiology.

## Materials and Methods

### *In silico* molecular dynamics simulations

Molecular dynamics simulations were performed as described by Mingo et al. using GROMACS v2020.4 and the monomeric structure of NSP2_WT_ (PDB#1L9V) or a NSP2_K294E_ model, which was created using UCSF Chimera (29, 44–46). The PDB file of the modeled structure is available upon request. Prior to performing the simulations, the structures were explicitly solvated with a three-point water model (TIP3P) in rhombic dodecahedron water box (solute-box distance of 1.0 nm) under periodic boundary conditions with charges neutralized by chloride ions. The AMBER99SB-ILDN force field was used for all simulations (47). Starting structures were energy minimized until convergence at Fmax<1000 kJ/mol/nm. A 100-picosecond position-restrained NVT equilibration simulation was run for water relaxation at 300K using a modified Berendsen (velocity rescaling) thermostat, followed by a 100-picosecond NPT equilibration simulation using the Parrinello-Rahman barostat for pressure coupling. After equilibration, an unrestrained 40-nanosecond NPT molecular dynamics simulation was run at 300K. Three trajectories initiated with different random seeds were run for each NSP2 structure. The RMSF of Δ-carbons from each of the three trajectories was calculated using the gmx rmsf command in GROMACS. B-factors (i.e., Debye-Waller factors) for each residue were calculated from the RMSF values using an established equation [B-factor = (8^2^/3) x (RMSF)^2^]. The averages of three trajectories were calculated, and one-tailed tests were performed, with *p* values <0.05 considered to be statistically significant. The three trajectories for each variant were concatenated in GROMACS and a cluster analysis performed with a cutoff of 0.2 nm using the gromos algorithm to allow examination of the structures most populated in the simulations as described in Nilsson et al. (48).

### Cell Culture

Monkey kidney cells MA104 (CRL-2378.1) or Cos-7 (CRL-1651) cells were obtained from American Type Cell Culture and sub-cultured in Medium 199 (M199) or Dulbecco modified Eagle medium (DMEM), respectively. Medium was made complete by supplementing to contain 100 U/ml penicillin-streptomycin (Gibco), 0.5 g/ml amphotericin B (Life Technologies), and 5% heat-inactivated fetal bovine serum (FBS) (Atlanta Biologics). Baby Hamster Kidney cells expressing the T7 polymerase (BHK-T7) were a kind gift from Dr. John Patton (Indiana University-Bloomington, IN), originating from the laboratory of Dr. Peter Collins (Laboratory of Infectious Diseases, NIAID, NIH, Bethesda, MD). BHK-T7 cells were grown in DMEM (Gibco), which was made complete by supplementing to contain 10% tryptone-peptide broth (Gibco), 100 U/ml penicillin-streptomycin, 2% non-essential amino acids (Gibco), 1% glutamine (Gibco), and 5% heat-inactivated FBS. The media used to cultivate BHK-T7 cells was also supplemented to contain 1 mg/ml G418 (Invitrogen) during every other cell passage (37). Culture media containing all supplements but lacking FBS (i.e., serum-free media) was used for RV infections. Cells were grown in a Thermo Forma 3110 CO_2_ water jacketed incubator at 37°C and split at 1:5 or 1:10 dilutions for <30 passages.

### Plasmid-based reverse genetics

Plasmids pT7-VP1-SA11, pT7-VP2-SA11, pT7-VP3-SA11, pT7-VP4-SA11, pT7-VP6-SA11, pT7-VP7-SA11, pT7-NSP1 -SA11, pT7-NSP2-SA11, pT7-NSP3-SA11, pT7-NSP4-SA11, and pT7-NSP5-SA11 as well as the support plasmid expressing the p10-FAST fusion protein, pCAG-FAST were developed by Kanai et al. and purchased from AddGene (37). The capping enzyme support plasmid, pCMV-NP868R, was kindly provided by Dr. John Patton (Indiana University, Bloomington, IN) (49). The complete sequences for strain SA11 genes are available in GenBank via accession no. LC178564-LC178574 (37). All plasmids were propagated in *E. coli* strain DH5a cells and purified using the Wizard Miniprep kit (Promega), according to the manufacturer’s protocol.

To generate the pT7-NSP2_K294E_-SA11 plasmid, site-directed mutagenesis was performed by outward PCR. Accuprime *Pfx* Supermix (Invitrogen) was used as the enzyme according to the manufacturer’s instructions, and the sequence was amplified using 5’-phosphorylated primers. PCR products were treated with *Dpnl* (New England Biolabs) to remove methylated template, and cDNAs were gel purified prior to their ligation using T4 DNA ligase (New England Biolabs) and subsequent transformation into *E. coli*. Plasmids were purified and subjected to Sanger sequencing (ETON Biosciences) across the entire genome segment cDNA to verify the introduction of the specific K294E change and the absence of additional mutations. Primers used for mutagenesis and sequencing are available by request.

The reverse genetics protocol was performed as described by Kanai et al. with minor modifications (37). BHK-T7 cells were seeded in 6-well plates (1.0 X 10^5^ cells per well) and grown for 48 h at 37°C. The cells were transfected with a reaction mixture containing 19.2 μl TransIT-LT1 (Mirus) in 250 μl of OptiMEM (Gibco), 0.8 μg each of the 11 pT7 plasmids, 0.8 μg of pCMV-NP868R, and 0.015 μg pCAG-FAST. To rescue the rRV-NSP2_K294E_ virus, the engineered pT7 construct (pT7-NSP2κ294E-SA11) was used *in lieu* of the wildtype construct. As a negative control, cells were transfected with all the plasmids except pT7-NSP2-SA11. All transfection reactions and subsequent rescue steps were performed in experimental duplicate. The transfected BHK-T7 cells were incubated at 37°C for 24 h, and then the culture media was changed to serum-free DMEM. The cells were incubated for an additional 24 h at 37°C prior to being overlayed with 1.0 X 10^5^ MA104 cells/well in serum-free DMEM supplemented to contain 0.5 μg/μl of porcine pancreatic type IX trypsin (Sigma Aldrich) to allow recombinant viral spread. The cells were incubated for 3 days at 37°C prior to being lysed by three rounds of freeze-thaw. The lysate (~2 ml total volume) was clarified by centrifugation at 1,500 X *g* for 5 min. Thereafter, ~1 ml of the clarified lysate was activated using 10 μg/μl porcine pancreatic type IX trypsin for 1 h at 37°C and used to infect MA104 cells (5 X 10^5^ cells) in well of a 6-well plate. The inoculum was adsorbed onto cells for ~1 h at 37°C, after which time it was replaced with serum-free M199 containing 0.5 μg/μl of porcine pancreatic type IX trypsin. Cells were incubated at 37°C for 3-5 days. Robust cytopathic effect (CPE) was observed for rRV-WT at 3 days p.i., so the cells were subjected to three rounds of freeze-thaw, and the clarified supernatant was stored as passage 0 (P0) stock. For rRV-NSP2_K294E_, no CPE was observed by 5 days p.i., and therefore, the cells were subjected to three rounds of freeze-thaw, and 1 ml of this clarified lysate was used to again infect MA104 cells as described above. After this second round of amplification, CPE was observed by 2 days p.i., and clarified lysate was stored as P0 stock. To create a passage 1 (P1) seed stock, 1 ml of each P0 stock was used to infect ~5 X 10^5^ cells MA104 cells in 25-cm^2^ flasks (5 ml volume) for 3 days at 37°C. To create a passage 2 (P2) working stock, 500 μl of the P1 stocks were used to infect 5 X 10^7^ MA104 cells in 175-cm^2^ flasks (20 ml volume in each flask) for 48 h at 37°C. The titers of the P2 working stocks were quantified by plaque assay at 37°C as described by Arnold et al., and representative plaque assay wells were photographed using a Amersham 600 gel imaging system (50). All viral stocks were aliquoted and stored at -20°C.

### RT-PCR and sanger sequencing

To verify the identity of the rescued viruses and ensure the absence of non-engineered mutations in the NSP2-coding genes, total RNA was extracted from 2 ml of the P2 working stocks for both rRV-WT and rRV-NSP2_K294E_ using TriZol™ (Invitrogen) according to manufacturer’s instructions. The NSP2-coding genes were amplified into full-length cDNAs using the Qscript XLT RT-PCR kit (Quanta BioDesign). Amplification products were purified using QIAquick PCR Purification Kit (Qiagen) and subjected to Sanger sequencing (ETON Biosciences). The complete NSP2 ORFs were fully sequenced with overlapping coverage at all positions. Contigs were assembled *de novo* by compilation of sequencing data in Geneious Pro v6.8.1 (Biomatters, Inc.) and compared to the sequence of the pT7-NSP2-SA11. Primers used for RT-PCR and sequencing are available upon request.

### Electropherotyping

Viral dsRNA was extracted from the P2 working stocks of SA11-4F (control), rRV-WT, and rRV-NSP2_K294E_ using Trizol according to the manufacturer’s instruction and resolved in a 4-15% SDS-polyacrylamide gel. The dsRNA was detected using SYBR Gold Stain (Invitrogen) according to the manufacturer’s instructions. Gels were imaged using Amersham 600 gel imaging system for fluorescence.

### Single-cycle replication assays

To characterize the growth phenotype of rRV-NSP2_K294E_ as compared to rRV-WT, single-cycle replication assays were performed in MA104 cells at 37°C. Viral P2 stocks were activated using 10 μg/μl porcine pancreatic type IX trypsin for 1 h at 37°C, followed by infection of MA104 cells (~5 X10^6^ cells/well) at a MOI of 3 PFU per cell. The inoculum was adsorbed for 1 h at 37°C and then replaced with complete M199. Infections proceeded at 37°C for 0, 12, or 24 h. The cells were lysed by three rounds of freeze-thaw, the supernatant was clarified by centrifugation at 1,500 X *g* for 5 min, and virus was quantified by plaque assay (50). Titers at the 12 and 24 h timepoints were normalized to the 0 h timepoint. The experiment was performed twice in experimental triplicate (n=6). Two-tailed tests were performed, and *p* values <0.001 were considered statistically significant.

### NTPase assays

The SA11 NSP2_WT_ ORF with a C-terminal hexahistidine tag (cHis) was subcloned from the pQE-60 vector into the pET-28a vector using 5’ *NcoI* and 3’ *HindIII* restriction sites (31). A thrombin cleavage site (amino acids LVPRGS) was engineered into the plasmid using site-directed mutagenesis and outward PCR to create pET-28a-NSP2wτ. Site-directed mutagenesis and outward PCR were then used to introduce nucleotide changes in the NSP2 gene corresponding to a K294E change in the NSP2 protein. Accuprime *Pfx* Supermix (Invitrogen) was used as the enzyme according to the manufacturer’s instructions, and the sequence was amplified using 5’-phosphorylated primers in both cases. PCR products were treated with *Dpnl* (New England Biolabs) to remove methylated template, and cDNAs were gel purified prior to their ligation using T4 DNA ligase (New England Biolabs) and subsequent transformation into *E. coli*. Plasmids were purified and subjected to Sanger sequencing (ETON Biosciences) across the entire genome segment cDNA to verify the introduction of the specific changes and the absence of additional mutations. Primers used for mutagenesis and sequencing are available by request.

The engineered pET-28a-NSP2wτ and pET-28a-NSP2_K294E_ vectors were used to express cHis-tagged NSP2_WT_ or NSP2_K294E_ proteins, respectively in *E. coli* Rosetta II cells according to the manufacturer’s protocol. Bacteria were grown 37°C in a shaking incubator until reaching to an optical density (OD_600_) of 0.4, at which point isopropyl β-D-1-thiogalactopyranoside was added to a final concentration of 1 mM to induce NSP2 expression. Following a 4-hour induction at 37°C, bacteria were harvested via centrifugation at 3836 x g for 15 min at 4°C. Pelleted bacteria were resuspended in lysis buffer (50 mM NaH_2_PO_4_, 400 mM NaCl, 20 mM Imidazole, 0.1% Triton X-100) containing EDTA-free protease inhibitor mini tablets (Pierce). Next, bacteria were lysed via sonication and centrifuged at 33, 600 x g for 15 min. The clarified lysate was incubated with HisPur cobalt resin (Pierce) while rocking for 2 h at 4°C. Unbound proteins were removed by three rounds of incubation in wash buffer (50 mM NaH_2_PO_4_, 500 mM NaCl, 20 mM Imidazole). The NSP2 proteins were eluted with elution buffer (50 mM NaH_2_PO_4_, 500 mM NaCl, 250 mM Imidazole) and exchanged into low salt buffer (20 mM Tris-HCL pH 7.5, 0.5 mM EDTA) using Amicon Ultra-4 centrifugation filters (Millipore). The purity and concentration of the resulting protein solution were assessed via electrophoresis in 10% SDS-PAGE gel alongside bovine serum albumin standards. The cHis tag was removed from an aliquot of each NSP2 protein using a Thrombin Cleavage Capture Kit (Millipore) following manufacturers guidelines. Successful removal of the cHis tag was confirmed by SDS-PAGE. Purified proteins were stored at -20°C and used within 4 days of purification.

The NTPase assay was adapted from that previously reported (31). A 50-μl reaction mixture was programmed to contain either no enzyme or 1 μg of NSP2 protein along with 50 mM Tris-HCl (pH 7.5), 5 mM MgCl^2^, and 50 μCi of [α^32^P]-ATP (3000 Ci/mmole). Aliquots of the reaction were collected at 0, 20, 40, and 60 min. The reactions were quenched by phenol:chloroform extraction, and the [α^32^P]-ADP product of hydrolysis was resolved from input [α^32^P]-ATP on PEI Cellulose F TLC plates (Millipore). Radiolabeled spots on the TLC plates were detected and quantified with a phosphoimager (Amersham Typhoon, GE) using the ImageQuant 5.0 software. P*i* levels were calculated by measuring the [α^32^P]ADP intensity /([α^32^P]ATP intensity + [α^32^P]ADP intensity) and were adjusted to the no enzyme control signals. The experiment was performed three times in experimental duplicate with at least two batches of purified NSP2 proteins.

### Immunofluorescence time course assays

To visualize viroplasms, MA104 cells were grown to confluency (5 X 10^5^ cells) on 18-mm glass coverslips in 12-well plates. Cells were either mock infected or infected at an MOI of 3 PFU per cell with trypsin-activated rRV-WT or rRV-NSP2_K294E_ for 1 h at 37°C. Inoculum was replaced with complete M199, and infections proceeded for 4, 8, 12, 16, or 24 h at 37°C. Coverslips were washed once with Dulbecco’s Phosphate Buffered Saline (DPBS) prior to being fixed and permeabilized by incubation in 100% methanol for 5 min at room temperature. To stain for NSP2, cells on coverslips were incubated overnight at 4°C in DPBS-T (DPBS, 0.1% Triton-X-100) supplemented with 5% bovine serum albumin (BSA) (EMD Millipore). Coverslips were then incubated with primary guinea pig αNSP2 antiserum (#2810) at a 1:1000 dilution in DPBS-T supplemented with 2.5% BSA for 1 h at room temperature with rocking. Cells were washed three times in DPBS-T and then incubated for 1 h at room temperature with goat anti-guinea pig Alexa 546 antibody (Life Technologies, Cat# A11074) at a 1:10,000 dilution in DPBS-T supplemented with 2.5% BSA. Nuclei were stained using Hoechst (Thermo, Cat # 62249) according to the manufacturer’s instructions. Coverslips were then mounted onto glass microscope slides using Prolong Glass Glass Mountant (Invitrogen, Cat# P36982).

Stained and mounted slides were imaged using a Zeiss LSM 880 inverted confocal microscope through a Plan-Apo 40xW/1.1 NA objective with distilled deionized water. The 405-nm and 561-nm laser lines were used, and sequential acquisitions were made to minimize cross-excitation. Images were collected at the optimal Z-plane at the highest pixel resolution. Images were processed and prepared for figures using Adobe Photoshop and Illustrator software. Three individual coverslips for each virus were blinded and quantified to determine the number and sizes of viroplasms. Specifically, at least 250 viroplasms and 25 cells per coverslip for each virus at each timepoint were quantified for a total of 750 viroplasms and 75 cells. Analysis was performed in FIJI using the original .czi files (11.122 pixels/micron) with the multi-point tool to count the number of viroplasms per cell, and the straight-line tool to measure the diameter of viroplasms. Raw data were binned using a custom K-Means R script, and bar graphs were generated in R. Statistical comparisons among counts were made with the negative binomial model and a dispersion parameter of 4.86, and *p* values <0.05 were considered statistically significant. Familywise error rates for the p-values resulting from multiple comparisons were corrected by the step-down method implemented in the multcomp package in the open-source statistical environment R. Statistical comparisons among diameters were made on Box-cox transformed data (implemented by the R package MASS using the optimal lambda parameter of 0.158) using a linear regression, and *p* values <0.05 were considered statistically significant. Familywise error rates for p-values from multiple comparisons were corrected as previously described.

### Western blotting

MA104 cells were grown to confluency (~5 X 10^5^ cells) in 6-well plates. Cells were either mock infected or infected at an MOI of 3 PFU per cell with trypsin-activated rRV-WT or rRV-NSP2κ294E for 1 h at 37°C. Inoculum was replaced with complete M199, and infections proceeded for 8 or 24 h at 37°C. Cells were harvested by scraping in 500 μl of lysis buffer (50 mM Tris pH 8.0, 150 mM NaCl, 1% NP-40, 0.5% DOC) and clarified by centrifugation at 16,873 x *g* for 5 min. Approximately 20 μl of each lysate was separated in 4-15% SDS-polyacrylamide gels, and proteins were transferred onto a nitrocellulose membrane (BioRad). The membranes were incubated in blocking solution (5% Carnation Instant Milk in Tris-buffered saline solution supplemented with 0.1% Tween (TBS-T)) overnight at 4°C. The membranes were then probed with a primary antibody: (i) mouse αtubulin (Invitrogen, Cat#5013044), (ii) guinea pig αNSP2 (2810), (ii) guinea pig αNSP5 (53964), or (iv) guinea pig αopen core (i.e., αVP1/VP2) (53961) at 1:500-1:1000 dilution in blocking solution for 1 h at room temperature. Following this, membranes were washed 3 times in TBS-T and incubated with anti-HRP conjugated secondary antibody (Invitrogen, Ref# 62-6520) at a 1:10,000 dilution in blocking buffer for 1 h at room temperature. Membranes were developed using the Thermo Pierce™ ECL Western Substrate method and imaged using Amersham 600 gel imaging system for chemiluminescence. Infections and western blots were repeated 3 times.

### Live-cell imaging

Live-cell imaging of viroplasms was employed using a stable cell line expressing an mCherry tagged to the C-terminus of wildtype NSP2 (strain RF) (16). Cells were seeded to confluency in Ibidi 35 mm Quad μ-Dish with #1.5 polymer coverslip (~8.5 X 10^4^ cells/quad). Cells were infected with trypsin-activated rRV-WT or rRV-NSP2_K294E_ at an MOI of 3 PFU per cell for 1 h at 37°C. Inoculum was replaced with phenol-free Medium (Gibco) supplemented with 2.5% FBS. The infected dish was then transferred to a Zeiss LSM 880 confocal microscope, and the infection was allowed to proceed for 16 h in a sealed, 37°C humidified chamber hooked up to 5% CO_2_. Cells were imaged every 10 minutes using a Plan-Apo 40X/0.95 NA air objective and laser line 561 nm. Tiled images and 5 Z-slices were collected from each quadrant at the highest pixel resolution.

Analysis of live-cell movies was performed with Fiji using the Trackmate plugin.Viroplasms were identified with CLIJ2 Vornoi-Otsu-Labelling with sigma and outline both set at 1. Incorrect spots were filtered out with area, circularity, and contrast parameters. Tracks were determined using Trackmate LAP detector, and any incorrect tracks were manually fixed (51). To measure viroplasm speed, data was collected from three separate regions of interest, with at least 30 viroplasms per region. Speed was averaged for each experiment, and a two-tailed unpaired t-test was used to determine significance, where *p* values <0.05 were considered statistically significant. The measure time-to-fusion, viroplasms were tracked starting when at least two viroplasms developed inside the same cell and were based on how many 10-min frames of the live cell imaging experiment it took for the two viroplasms to merge, and 20 viroplasm merging events were analyzed per virus. A two-tailed unpaired t-test was used to determine significance, and *p* values <0.05 were considered statistically significant.

### Perinuclear ratio [V/C] assay

Fixed confocal micrographs obtained as described above (αNSP2 staining) were analyzed for perinuclear condensation using FIJI. The [V/C] ratio was determined as described previously by (13). Briefly, the total cell area (c), distribution of viroplasms (v), and nucleus area (n) boundaries were determined by hand-drawing boundaries using a Huion KAMVAS Pro 16 Graphics Drawing Tablet and the free-hand tool in FIJI. The measurement function was used to determine the area for each boundary. Subsequently, the condensation of either rRV-WT or rRV-NSP2κ294E viroplasms to the perinuclear area is expressed as [V/C], where V=v-n and C=c-n. A two-tailed unpaired T-Test was used to determine significance, and *p* values <0.05 were considered statistically significant.

### VLS formation assay

The NSP2_WT_ ORF (strain SA11) was subcloned into the pEGFP-N1 vector to allow for expression of NSP2_WT_ as a C-terminal fusion to enhanced GFP. Site-directed mutagenesis by outward PCR was performed to introduce mutations that would delete the NSP2 CTR residues 293-317 (for NSP2_ΔCTR_) or that would introduce the codon changes for K294E, K294A, and K294R (for NSP2_K294E_, NSP2_K294A_ or NSP2_K294R_). VLS formation was tested as described by McKell et al with some modifications (22). Briefly, Cos-7 cells were seeded on 18-mm glass coverslips in 12-well plates and grown to ~50% confluence. Approximately 0.5 X 10^6^ cells/well were transfected with 0.5 μg of pCI-NSP5 (22) and 0.5 μg of each pEGFP-N1 NSP2 construct using Trans-IT®-LT1 (Mirus) according to the manufacturer’s instructions. The transfected cells were incubated for 48 h at 37°C to allow for protein expression. Thereafter, coverslips were fixed and stained as described above for the infected cell immunofluorescence, except using primary guinea pig aNSP5 (53964) antiserum.

Stained and mounted slides were imaged using a Zeiss LSM 880 inverted confocal microscope through a Plan-Apo 40xW/1.1 NA objective with distilled deionized water. The 488-nm and 561-nm laser lines were used, and sequential acquisitions were made to minimize cross-excitation. Images were collected at the optimal Z-plane at the highest pixel resolution. Images were processed and prepared for figures using Adobe Photoshop and Illustrator software. Three individual coverslips for each clone were imaged and quantified in a blinded manner, where 30 different NSP2/NSP5 co-expressing cells were quantified per coverslip for a total of 90 cells quantified per NSP2 variant. The number of cells forming VLSs for the NSP2 CTR mutants (NSP2_ΔCTR_, NSP2_K294A_, or NSP2_K294R_) was calculated as a percentage of those formed with NSP2_WT_.

### *In vitro* LLPS droplet assay

Recombinant, cHis-tagged NSP2 proteins (NSP2_WT_ and NSP2_K294E_) and N-terminally StrepII-tagged NSP5 proteins were expressed and purified as described above and previously (16, 30). NSP2_WT_ and NSP2_K294E_ were labelled with NTA-Atto-488 and then mixed with purified unlabeled NSP5 at varying concentrations (5-20 μM) in DPBS (Sigma). To quantify droplet size, 15 μM of each protein were used. Specifically, 2 μl of the protein mixture was incubated on a clean 18-well glass bottom μ-Slide (Ibidi) for 5 min before imaging of phase diagrams and for 0 or 15 min before imaging for droplet quantification. Images were recorded using an ONI Nanoimager S microscope equipped with a sCMOS camera (pixel size of 0.117 μm) and an Olympus 100 x 1.4 NA oil immersion objective. An exposure time of 33 milliseconds was used with a laser power of 1%. An imaging region of 50 μm x 80 μm, 250 μm x 400 μm or 500 μm x 800 μm was scanned for Atto488 signal for generation of phase diagrams or the quantification of droplets at 0 or 15 minutes, respectively. Fiji (ImageJ) was used for image analysis and droplet quantification (52). Binary images were created using the thresholding method described by Yen et al., which allowed for automated particle analysis (53). At an incubation time of 0 minutes, 18 particles were counted for NSP2_WT_ and 10 for NSP2_K294E_. After an incubation of 15 minutes, 3568 particles were counted for NSP2_WT_ and 1461 particles for NSP2_K294E_. Statistical analyses data plotting was performed using GraphPad Prism (Version 9.3.1). An unpaired t-test was performed for both timepoints, and *p* values <0.005 were considered statistically significant.

## Importance

Viruses often condense the materials needed for their replication into discrete intracellular factories. For rotaviruses, agents of severe gastroenteritis in children, factory formation is mediated in part by an octameric protein called NSP2. A flexible C-terminal region of NSP2 has been proposed to link several NSP2 octamers together, a feature that might be important for factory formation. Here, we created a change in NSP2 that reduced C-terminal flexibility and analyzed the impact on rotavirus factories. We found that the change caused the formation of smaller and more numerous factories that could not readily fuse together like those of the wildtype virus. The altered NSP2 protein also had a reduced capacity to form factory-like condensates in a test tube. Together, these results add to our growing understanding of how NSP2 supports rotavirus factory formation—a key step of viral replication.
